# Exploring the differentiation of behavioural and emotional problems across childhood: A prospective longitudinal cohort study

**DOI:** 10.1002/jcv2.12176

**Published:** 2023-06-30

**Authors:** Adrian Dahl Askelund, Helga Ask, Eivind Ystrom, Alexandra Havdahl, Laurie J. Hannigan

**Affiliations:** ^1^ Department of Psychology University of Oslo Oslo Norway; ^2^ Nic Waals Institute Lovisenberg Diaconal Hospital Oslo Norway; ^3^ Center for Genetic Epidemiology and Mental Health Norwegian Institute of Public Health Oslo Norway; ^4^ Department of Psychology PROMENTA Research Center University of Oslo Oslo Norway; ^5^ MRC Integrative Epidemiology Unit Bristol Medical School University of Bristol Bristol UK

**Keywords:** behavioural problems, birth cohort, child development, emotional problems, health registry

## Abstract

**Background:**

An individual's overall burden of behavioural and emotional problems across childhood is associated with increased likelihood of later mental health conditions. However, the *relative* extent of behavioural versus emotional problems ‐ that is, the extent to which the domains are *differentiated* from one another ‐ may provide additional information about who is at risk of developing a mental health condition. Here, we seek to validate differentiation as an independent predictor of later mental health conditions, and to explore its aetiology.

**Methods:**

We analysed data from ~79,000 children in the population‐based Norwegian Mother, Father, and Child Cohort Study, and linked health‐care registries. In preregistered analyses, we modelled the extent and rate of differentiation of behavioural and emotional problems between ages 1.5–5 years, and estimated associations with later symptoms (age 8) and diagnoses (after age 8). We also explored the aetiology of differentiation by estimating associations with early life exposures and, in a subset of 23,945 full siblings, assessing the impact of accounting for unobserved familial confounding.

**Results:**

Differentiation of behavioural and emotional problems was associated with later symptoms and diagnoses of mental health conditions, independent of total problems. Maternal at‐risk drinking (*β* = 0.04 [0.02, 0.06]) and parental relationship problems (*β* = 0.04 [0.02, 0.05]) were associated with higher behavioural relative to emotional problems at age 5. Maternal prenatal distress (|β| = 0.04 [0.03, 0.06]), concurrent distress (|β| = 0.04 [0.02, 0.06]) and parental education (|β| = 0.05 [0.04, 0.07]) predicted higher emotional relative to behavioural problems at age 5. Estimates for maternal prenatal distress and at‐risk drinking were consistent across both unadjusted and adjusted analyses accounting for unobserved familial risk.

**Conclusions:**

Differentiation of behavioural and emotional problems in early childhood represents a valid source of inter‐individual variability linked to the later emergence of psychopathology and may be relevant for early detection and prevention strategies for mental health.

**Key Points**

- Co‐occurring behavioural and emotional problems in early life are strongly associated with later mental health conditions. However, the relative extent of problems in these domains (i.e., differentiation) may provide additional information about who is at risk and for which disorders, essential to detecting and preventing the emergence of psychopathology.
- Findings show that differentiation of behavioural and emotional problems in early life strongly associates with the development of mental health conditions later in childhood and adolescence.
- We identify potentially modifiable environmental factors predicting differentiation in early life, while accounting for confounding by unobserved familial risk.

## INTRODUCTION

Behavioural and emotional problems often co‐occur in young children. It is also well established that behavioural problems (i.e., undercontrolled and disruptive behaviour) and emotional problems (i.e., negative mood states and inhibition) in early childhood are associated with an increased risk of mental disorders later in development (Caspi et al., 1996; Mulraney et al., 2021). While common, the co‐occurrence of behavioural and emotional problems is not universal. Many children display behavioural problems without concurrent emotional problems, while others exhibit no behavioural problems despite considerable emotional problems. The relative extent of children's difficulties in these domains ‐ that is, the extent to which behavioural and emotional problems are *differentiated* from one another ‐ may provide additional information about who is at risk of developing specific mental health conditions.

When seeking to understand the relationship between childhood behavioural and emotional problems and later mental disorders, researchers have typically taken one of two approaches. Either behavioural and emotional problems are considered separately (emphasising their distinctiveness, i.e., Lund et al., 2020), or they are combined into a single dimension (emphasising their overlap, i.e., Scott et al., 2009). Comparatively little research has focused explicitly on the extent of differentiation between these domains; that is, inter‐individual variability in the concordance between behavioural and emotional problems. There are reasons to believe that this variability ‐ which is, by definition, unrelated to the total burden of problems an individual experiences ‐ may provide important additional information about the risk for specific, future psychiatric problems. First, clinicians rely on distinct profiles of symptoms to assign diagnoses. This means that, while having a high burden of problems across domains may increase an individual's likelihood of presenting for clinical diagnosis, the specific and relative pattern of their symptoms will inform which diagnosis they receive. Second, decreasing correlations among mental health conditions as children grow older may be indicative of differentiation. Previous studies have shown that the largest decreases in correlations among pairs of mental health conditions were between those belonging to the behavioural and emotional domains rather than within either domain (Sterba et al., 2010). Third, previous research has found that when a general psychopathology (“p”) factor is accounted for, specific behavioural and emotional problem factors are associated with problems in a wide range of domains (Caspi et al., 2014). Finally, even without explicitly defining differentiation of emotional and behavioural problems, studies showing independent or heterogeneous associations between these domains and later mental health outcomes (Mulraney et al., 2021) provide indirect evidence that the predictive capacity of overall problems in childhood for such outcomes is more than the sum of its parts.

Insofar as differentiation of behavioural and emotional problems in childhood proves to be independently predictive of later mental health outcomes, exploring its aetiological basis will be important for informing early detection and prevention strategies. Observational studies have identified associations between various early environmental risk factors and child behavioural and emotional problems, including—but not limited to—socioeconomic disadvantage (Caspi et al., 2016; Costello et al., 2003; Dearing et al., 2006; Leventhal & Dupéré, 2019), maternal depression (Gjerde et al., 2021; Goodman et al., 2011), adverse childhood experiences (Liming & Grube, 2018), parental disharmony (Auersperg et al., 2019; Jenkins & Smith, 1991), and parental smoking and at‐risk drinking (Brion et al., 2010; Lund et al., 2020). It is likely, though not yet established, that similar factors are involved in differentiating behavioural and emotional problems in early childhood. Given the expectation that the extent of differentiation of behavioural and emotional problems may increase over time, it is notable that behavioural genetic evidence provides consistent support for the notion that non‐shared environmental factors ‐ to a greater degree than genetic factors ‐ influence developmental change in behavioural and emotional problems (Hannigan et al., 2017).

To explore the aetiological underpinnings of differentiation of behavioural and emotional problems in childhood, it is essential to consider possible routes of confounding. Both childhood behavioural and emotional problems (Polderman et al., 2015) and measures of the childhood environment (Kendler & Baker, 2007) are influenced by genetic factors. Since parents provide both genes and environments to their children, this leads to confounding by gene‐environment correlations (Plomin et al., 1977). Therefore, it is crucial to study early environmental risk factors while appropriately accounting for potential confounding due to unobserved familial risk. While recently developed methods offer ways of doing this via the incorporation of parental genetic data (Cheesman et al., 2020; Eilertsen et al., 2022), the most powerful designs for partitioning out covariation attributable to confounding influences on childhood exposures and outcomes continue to rely on structured family data. Foremost among these are adoption (Kendler et al., 2022) or assisted conception (Rice et al., 2013) designs, wherein the separate influences of biological and rearing parents can be conclusively delineated. However, the sibling comparison design is a powerful alternative. In this design, information ​​from siblings with discordant exposure to putative environmental risks, and/or differential scores on outcomes, can be used to estimate an exposure‐outcome association free of unobserved familial confounding. Although models based on sibling comparisons are not without limitations ‐ including risks of biases being induced as well as removed (Frisell, 2021) ‐ comparing unadjusted and adjusted estimates from these models can provide vital context with which to interpret exposure‐outcome associations. Sibling comparison models, and other designs equipped to partition out familial confounding, have been widely used to study associations between early life exposures and childhood mental health (for a recent review, see Jami et al., 2021), but not previously with a specific focus on uncovering which exposures may influence how behavioural and emotional problems differentiate from one another across development.

Here, we extend earlier studies of differentiation in childhood by investigating its links to early life exposures in models that account for common sources of shared confounding. We propose a novel operationalisation of differentiation as a developmental process: that is, each person's relative level of, and rate of change in, behavioural versus emotional problems during early childhood. We seek to validate that differentiation operationalised in this way is a relevant factor in the developmental emergence of psychopathology, by establishing whether it is associated with symptom scores in middle childhood, and registry‐based diagnoses of mental health conditions in later childhood and adolescence, independently of total problems. Next, we explore associations between early life exposures and differentiation across childhood, looking for attenuation in effects estimated within sibling pairs as an indicator of the influence of biases from unobserved familial confounding.

## MATERIALS AND METHODS

### Design

#### Sample

We used data from a population‐based sample of children from the Norwegian Mother, Father, and Child Cohort Study (MoBa; Magnus et al., 2016, 2006) conducted by the Norwegian Institute of Public Health. Participants were recruited from all over Norway from 1999 to 2008. The women consented to participation in 40.6% of the pregnancies. The cohort now includes 114.500 children, 95.200 mothers and 75.200 fathers.

The analyses were based on version 12 of the quality‐assured data files released for research in January 2019. We also used data from the Medical Birth Registry of Norway (MBRN), a national health registry containing information about all births in Norway. In addition, we obtained data on diagnoses of specific mental health conditions from the Norwegian Patient Registry (NPR) and “Kontroll og utbetaling av helserefusjoner” (KUHR).

The data in MoBa was collected by questionnaires from early pregnancy to middle childhood, provided primarily by mothers (around week 17 and 30 of pregnancy, when the child was 6 and 18 months, and at 3, 5, and 8 years). Phenotype data from the fathers were collected by questionnaire around week 17 of the pregnancy.

#### Measures

##### Differentiation of behavioural and emotional problems

**FIGURE 1:**
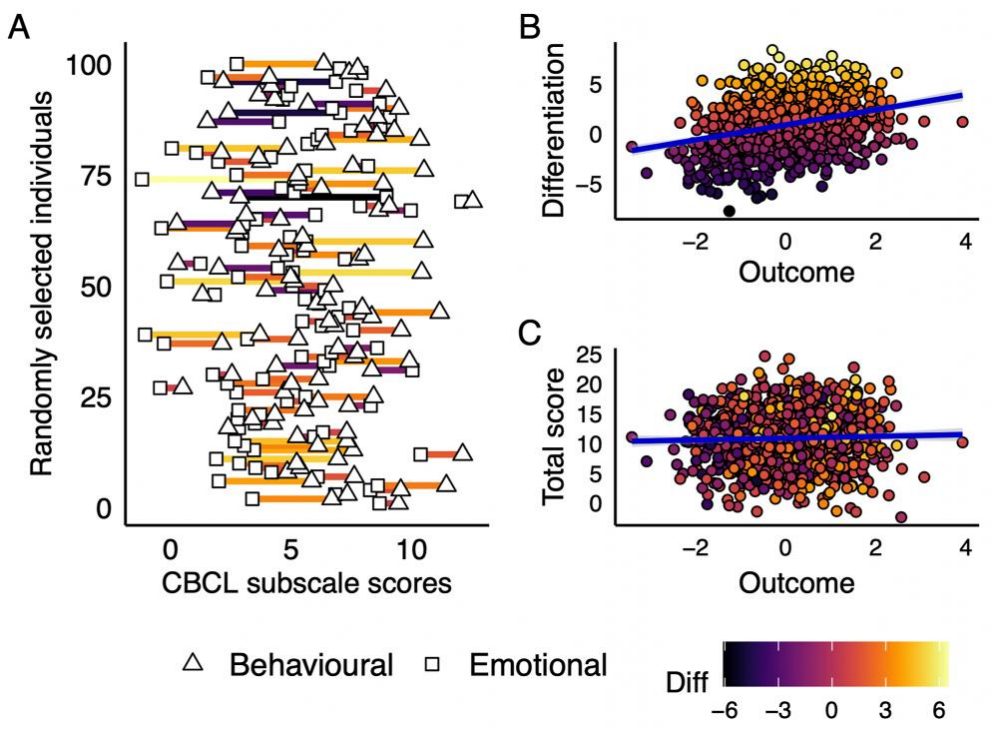
Operationalisation and properties of CBCL differentiation/total scores. Panel A shows how the differentiation score is constructed based on individual scores on the behavioural and emotional subscales of the CBCL in 100 randomly selected individuals from a simulated dataset (*N* = 1000; *r*_*beh_emo*_ = 0.5, *M*_*emo*_ = 5, *M*_*beh*_ = 6, *SD*_*emo/beh*_ = 2.5); B and C show that since the computed scores are orthogonal to one another, it is possible for the differentiation score (in B) to be associated with an outcome which the total score (in C) is not; CBCL, Child Behaviour Checklist.

We used the Child Behaviour Checklist (CBCL) to assess behavioural problems (8 items) and emotional problems (5 items), at ages 1.5, 3, and 5 years. Information about psychometric properties of the CBCL subscales are provided in Appendix S1 and Table S1. Differentiation was operationalised as the difference between standardised scores of behavioural and emotional problems (behavioural problems ‐ emotional problems = difference score; see Figure 1, panels A/B for an illustration). This means that individuals with high difference scores have relatively more behavioural than emotional problems, while individuals with low scores have the inverse.

##### Total behavioural and emotional problems

We also combined behavioural and emotional problems as a sum score in order to provide a point of reference for analyses of the differentiation scores described above. Total levels of emotional and behavioural problems were computed by adding up standardised scores of the two dimensions in the overall sample (behavioural problems + emotional problems = total score). By definition, these scores are completely orthogonal to the differentiation scores.

##### Symptoms of mental health conditions at 8 years

We used the 13‐item Short Mood and Feelings Questionnaire (SMFQ; Angold et al., 1995) to assess symptoms of depression, a 5‐item version of the Screen for Child Anxiety Related Disorders (SCARED; Birmaher et al., 1997) to assess symptoms of anxiety, and a 34‐item version of the Rating Scale for Disruptive Behaviour Disorders (RS‐DBD; Silva et al., 2005) to assess symptoms of hyperactivity and inattention (subscales related to attention‐deficit hyperactivity disorder [ADHD], 18 items), conduct disorder (CD, 8 items), and oppositional defiant disorder (ODD, 8 items). The measures were treated as continuous, and scores were standardised to have a mean of 0 and standard deviation of 1. Information about psychometric properties of all symptom scales at age 8 are in Appendix S1 and Table S2.

##### Diagnoses of mental health conditions

We linked to KUHR and NPR to obtain diagnoses of mental health conditions from medical records (depression, anxiety, ADHD, and disruptive behaviour disorders [DBD], combining CD and ODD; see Appendix S2 for information about diagnostic codes). Individuals were classified as a “case” if they had received a relevant diagnosis at least twice in either primary or secondary health care, or once in each, between their eighth birthday and July 2021, the end of the available follow‐up.

##### Early life exposures

We included measures of parental income and education, and both mother‐ and father‐reported adverse life events, mental distress, relationship problems, smoking and at‐risk drinking, and maternal alcohol problems and postnatal depression as predictors. A full overview of variables, including information about variable processing, is in Table S3. All deviations from the preregistered list of early life exposures, and reasons for these deviations are described in Appendix S3.

##### Covariates

We included the child's sex (as registered at birth in MBRN) and parity as time‐invariant covariates and the child's age at questionnaire return as a time‐varying covariate. Mother ID was used for clustering in all models.

### Statistical analyses

All modelling was carried out in R version 4.1.2, including analyses requiring Mplus (Muthén & Muthén, 1998), through the MplusAutomation package version 1.0.0 (Hallquist & Wiley, 2018). The phenotools package version 0.2.8 was used to process questionnaire and registry data (https://github.com/psychgen/phenotools). We used the False Discovery Rate (FDR) to preserve the Type 1 error rates at 5% in all models (Benjamini & Hochberg, 1995). We used a maximum likelihood estimator which computes robust standard errors and a scaled test statistic.

#### Modelling differentiation as a developmental process

Using latent growth modelling (LGM), we parameterised the development of behavioural‐emotional differentiation across three time points (when the children were 1.5, 3, and 5 years of age). Individual differences in the extent and rate of differentiation of behavioural and emotional problems were captured by latent slope and intercept factors (see Figure S1 for an illustration of this model). The intercept was set at 5 years, as we were primarily interested in the endpoint of the children's differentiation process. We also ran equivalent LGMs of total childhood behavioural and emotional problems. In all models we accounted for dependencies in the data (due to participating siblings) by clustering within mothers.

#### Validation of differentiation

To validate our operationalisation of differentiation, we ran LGMs where both the slope and intercept, and each growth factor individually, were allowed to influence 8‐year symptoms of depression, anxiety, hyperactivity, inattention, CD, and ODD. Similarly, we explored associations between differentiation and individuals' likelihood of having a recorded diagnosis of depression, anxiety, ADHD, and DBD, using extracted factor scores as predictors of diagnostic outcomes in a logistic regression framework. To assess the extent of bias arising from non‐random non‐participation in MoBa, we conducted sensitivity analyses using inverse probability of participation weighting based on aggregated statistics on parity and cohabitation status from Statistics Norway (see Supporting Information Appendixes for further details).

#### Investigating predictors of differentiation

We entered the 16 early life exposures into the LGMs, initially allowing them to influence both slope and intercept factors to derive estimates of the observational exposure‐outcome associations in the full sample. We then tested sub‐models in which all exposures influenced the growth process via, alternately, only the slope or intercept factors. Finally, we restricted to the sibling sub‐sample (23,945 full siblings) and ran multilevel LGMs, where variance in the exposure variables is decomposed into variance that is specific to a given mother for a given child (level 1) and variance that is specific to a given mother but shared across siblings (level 2). Effects of the exposures on the growth factor(s) were estimated in two versions of this model: (1) unadjusted for familial confounding (equivalent to the observational exposure‐outcome associations run in the full sample) and (2) adjusted for nuclear family‐level confounding. Whilst only controlling for 50% of genetic effects at the child level, this design completely adjusts associations for family‐wide factors. This includes most plausible “third variable” confounders of associations between early environmental exposures and child outcomes (i.e., 100% maternal genetic and stable environmental factors influencing predictors).

To account for differences between the sibling sub‐sample and the overall sample, due to within‐sample selection bias (parents of MoBa siblings, by definition, participate on more than one occasion), we included inverse probability weights based on a logistic regression of sibling‐singleton status on all other study variables in all sibling‐only models (see Supporting Appendix S5 for full details).

All analyses were again repeated for the total problems outcomes to contextualise the differentiation results.

#### Inference criteria

Likelihood ratio tests were used to select the best‐fitting LGMs. Effect sizes and confidence intervals were used to draw inferences about associations between exposures and outcomes in the full sample analyses. The relative attenuation of exposure‐outcome associations in the adjusted versus unadjusted multilevel models was used to guide inferences about the likely role of confounding in observational exposure‐outcome associations.

The following fit statistics were reported for each model: the comparative fit index (CFI), Tucker‐Lewis index (TLI), standardised root mean square residual (SRMR), and root mean square error of approximation (RMSEA). By convention, an SRMR below 0.1 and an RMSEA of less than 0.05 implies a good fit, as well as CFI and TLI values over 0.95 (Kline, 2011). We also reported the Akaike Information Criterion and Bayesian Information Criterion (BIC), for which lower values are preferred when comparing models. For nested models, −2LL based likelihood ratio tests were used to select the better fitting model. For non‐nested models, the one with the lower BIC was preferred.

### Inclusion criteria and sample size

We included all MoBa children with available CBCL data on at least one measurement occasion (see Table S4 for response numbers at each wave, and Appendix S4 for details on handling of outliers). The overall sample size was 78,982 (38,544 female), including 23,945 full siblings. Note that a more limited sample of 31,854 children had CBCL information on all waves, but we used a full information maximum likelihood approach, which includes all available data and yields unbiased parameter estimates assuming data is missing at random or missing completely at random (Enders & Bandalos, 2001).

### Availability of data and analytic code

The MoBa data are not publicly available as the consent given by the participants does not open for storage of data on an individual level in repositories or journals. Researchers who want access to data sets for replication should submit an application to datatilgang(at)fhi.no. Access to datasets requires approval from The Regional Committee for Medical and Health Research Ethics in Norway and an agreement with MoBa.

Data preparation and analysis code for all elements of the project is publicly available on Github: https://github.com/psychgen/childhood‐differentiation.

## RESULTS

Descriptive statistics for all symptom measures are presented in Table S4. There was evidence of a low level of selective attrition based on the CBCL subscales (see Appendix S6). As expected, the differentiation and total scores were empirically independent (*r*_range_: −0.01–0.01).

### Modelling differentiation as a developmental process

A linear growth model with both slope and intercept factors provided excellent fit to the data using the differentiation scores of behavioural and emotional problems from age 1.5–5 years (CFI = 0.99, TLI = 0.98, RMSEA = 0.01, SRMR = 0.01; see Table S5 for model comparisons). The correlation between the slope and the intercept was positive (*r* = 0.64 [0.62, 0.66]). There was evidence of sex differences where males showed greater extent of behavioural relative to emotional problems at age 5 (*β*_intercept_ = 0.09 [0.08, 0.10]) but similar rates of differentiation over time compared to females (*β*_slope_ = 0.01 [−0.01, 0.02]). Later birth order was associated with greater extent (*β*_intercept_ = 0.05 [0.04, 0.06]) and rate (*β*_slope_ = 0.06 [0.04, 0.07]) of differentiation toward behavioural problems. For total problems, a growth model with both slope and intercept factors also provided excellent fit (CFI = 0.99, TLI = 0.98, RMSEA = 0.02, SRMR = 0.01; see also Table S5).

### Validation of differentiation

**FIGURE 2:**
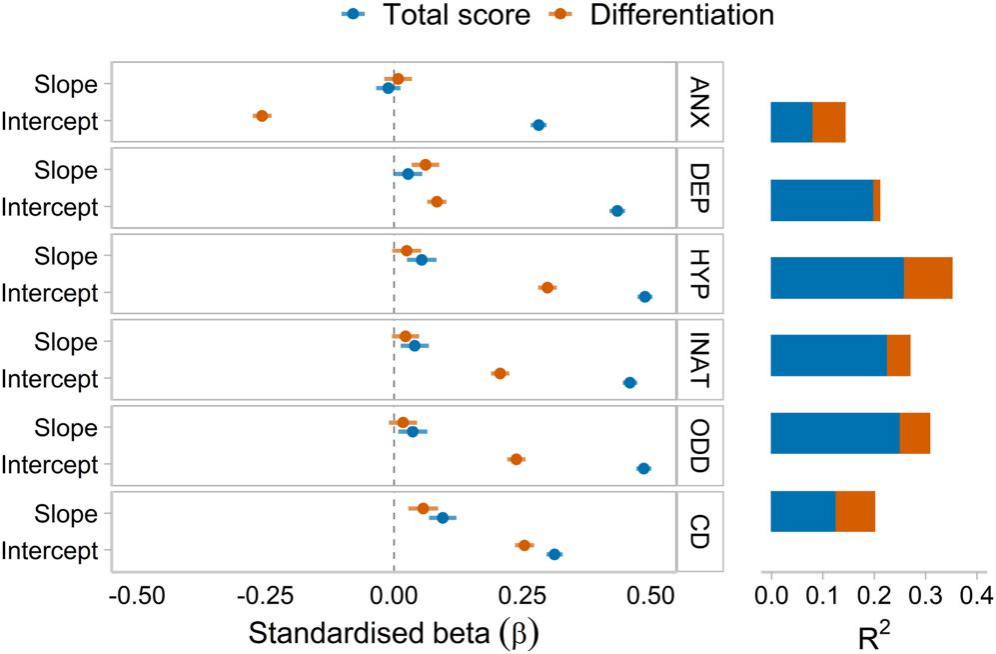
Results from the validation analysis showing that differentiation in early childhood predicts 8‐year symptoms over and above total levels of behavioural and emotional problems. Intercept and slope of differentiation and total problems predicting symptoms of mental health conditions at age 8; ANX, anxiety; CD, conduct disorder; DEP, depression; HYP, hyperactivity; INAT, inattention; ODD, oppositional defiant disorder; R^2^, R squared.

We tested the associations between differentiation and symptom domains at age 8 (see Figure 2). The best‐fitting models included effects from both intercept and slope factors to the 8‐year symptom outcomes (i.e., dropping effects led to significant decrement of fit, see Table S6). We observed associations between the slope and/or intercept of differentiation with all outcomes (Figure 2). Overall, differentiation towards behavioural relative to emotional problems in early childhood predicted higher symptoms of behavioural conditions in middle childhood (i.e., hyperactivity, inattention, CD, and ODD), and differentiation towards emotional problems predicted higher anxiety. For total problems, effects from both slope and intercept factors provided best fit (see Table S6), and associations with 8‐year symptoms were generally stronger (see Figure 2).

**FIGURE 3:**
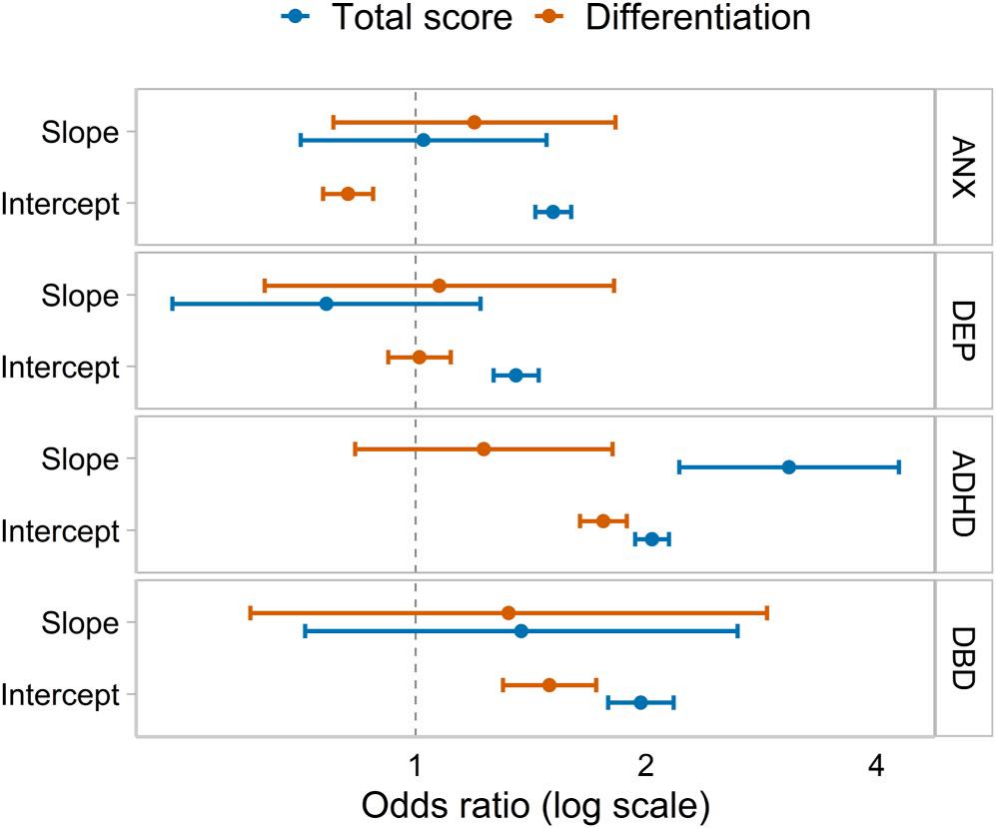
Validation analysis showing associations between differentiation and total problems in early childhood and odds of receiving diagnoses later in childhood and adolescence. Estimates were derived based on extracted factor scores, predicting diagnoses of mental health conditions (after age 8) in logistic regressions; ANX, anxiety; DEP, depression; ADHD, attention‐deficit hyperactivity disorder; DBD, disruptive behaviour disorders, including oppositional defiant disorder and conduct disorder.

We then estimated the likelihood of having received relevant diagnoses based on the rate and extent of differentiation during early childhood (see Figure 3). In these analyses, the largest associations were seen for the rate (OR_slope_ = 3.07 [2.21, 4.27]) and extent (OR_intercept_ = 2.04 [1.94, 2.14]) of total problems and the rate (OR_slope_ = 1.23 [0.83, 1.81]) and extent (OR_intercept_ = 1.76 [1.64, 1.89]) of differentiation toward behavioural problems in relation to a diagnosis of ADHD. Similar associations were observed between the extent of total problems (OR_intercept_ = 1.97 [1.78, 2.17]), differentiation toward behavioural problems at age 5 (OR_intercept_ = 1.50 [1.30, 1.72]), and later diagnosed DBD.

### Investigating predictors of differentiation

**FIGURE 4:**
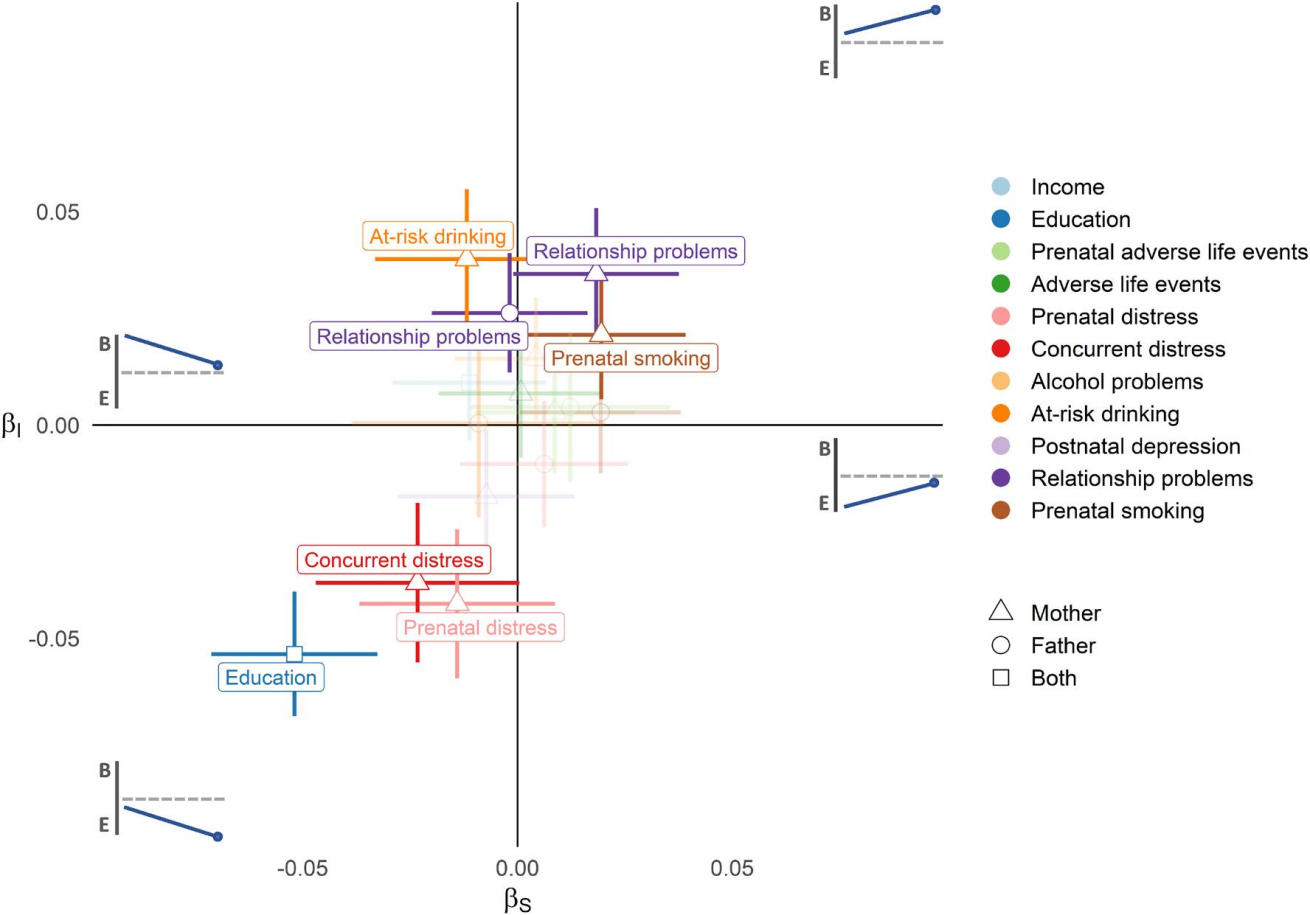
Observational results of early life exposures on the extent and rate of differentiation in childhood in the full sample. Standardised betas (+behavioural and ‐emotional) for the observational associations of predictors with the intercept (*β*_*I*_) and slope (*β*_*S*_) of differentiation; the upper left quadrant represents converging behavioural problems; the lower left quadrant represents differentiation toward emotional problems; the upper right quadrant represents differentiation toward behavioural problems; the lower right quadrant represents converging emotional problems; predictors that are labelled in the plot were significant after False Discovery Rate (FDR) correction, and predictors that are transparent did not; lines denote 95% confidence intervals; B, behavioural; E, emotional.

The best‐fitting model for differentiation included effects of early life exposures on both slope and intercept factors (see Table S7). In the full sample observational LGM, parental education predicted the extent (*β*_intercept_ = −0.05 [−0.07, −0.04]) and rate (*β*_slope_ = −0.05 [−0.07, −0.03]) of differentiation toward emotional problems. In addition, maternal prenatal and concurrent distress were predictive of the extent and/or rate of differentiation toward emotional problems after FDR correction (see Figure 4). The following predictors of the extent and/or rate of differentiation toward behavioural problems remained after FDR correction in the observational LGM: maternal prenatal smoking and parental relationship problems. In addition, maternal at‐risk drinking predicted a higher extent of behavioural problems at age 5 but similar rate of change over time. Parental education and maternal concurrent distress were the most important predictors of total problems (see Figure S2).

**FIGURE 5:**
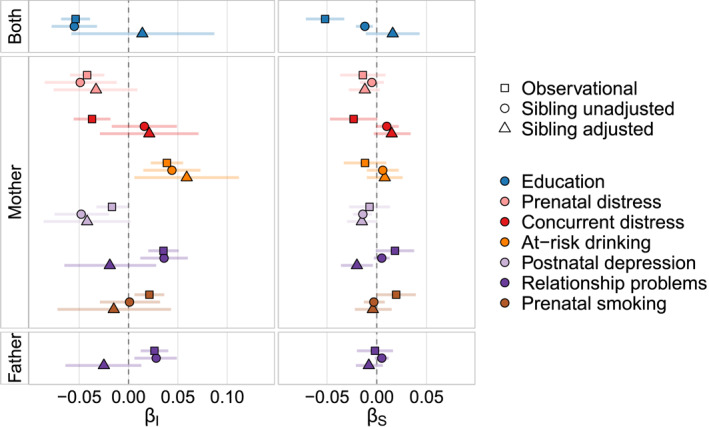
Full sample, sibling unadjusted and adjusted associations between early life exposures and differentiation in childhood. Standardised betas (−emotional and +behavioural) for observational (*N* = 78,982), as well as sibling adjusted and unadjusted (*N* = 23,945 full siblings) associations between predictors and the intercept (*β*_*I*_) and slope (*β*_*S*_) of differentiation; only False Discovery Rate (FDR) significant predictors from the observational analysis are shown here; note that the sibling adjusted results are imprecise due to the weighting procedure used to adjust for differences between siblings and singletons, meaning that attention should mainly be paid to the attenuation (or not) of the point estimates.

We then ran multilevel models in the sub‐sample of siblings, comparing the point estimates for exposure‐outcome associations before and after adjusting for confounding by unobserved familial risk (see Figure 5). Maternal at‐risk drinking predicted relatively higher behavioural than emotional problems consistently strongly in both unadjusted and adjusted models. Maternal postnatal depression also predicted relatively higher emotional than behavioural problems consistently strongly in both models, whereas the associations for parental education and relationship problems were attenuated. Effects were broadly attenuated for total problems, with parental education and maternal concurrent distress being attenuated to near zero (see Figure S3). Maternal adverse life events remained associated with higher total behavioural and emotional problems, whereas parental income seemed to emerge as a protective factor after adjustment.

To help contextualise the findings relating to differentiation, we also conducted sensitivity analyses with the CBCL behavioural and emotional problems subscales included as separate, correlated outcomes (results for behavioural problems are shown in Tables S9 and S10, and emotional problems in Table S11 and S12). This sensitivity analysis showed that maternal at‐risk drinking was associated with both higher behavioural problems *and* lower emotional problems, such that the relative distance between them made maternal drinking an important predictor of differentiation. Conversely, the adjusted effect for postnatal depression on differentiation appeared to be primarily driven by postnatal depression being associated with fewer behavioural problems.

## DISCUSSION

In summary, we explored the differentiation of behavioural and emotional problems across childhood using data from a large population‐based birth cohort. Supporting the validity of our approach, early‐life differentiation was associated with symptoms of mental health conditions in middle childhood and clinical manifestations of those conditions later in childhood and adolescence. We identified potentially modifiable parental factors associated with children's differentiation toward behavioural problems (e.g., maternal at‐risk drinking) and emotional problems (e.g., maternal prenatal distress). In contrast to observational links between differentiation and parental education or relationship problems, these associations appeared not to be inflated by unobserved familial confounding.

There has recently been much emphasis on the stability and co‐occurrence of mental health conditions in childhood, which can be captured by a general “p” factor (Allegrini et al., 2020; Lahey et al., 2015; Murray et al., 2016). However, it remains the case that specific components of liability to psychopathology not captured by this general factor are consequential (Caspi et al., 2014). Here, taking a statistically distinct but theoretically consistent approach to extracting this specific signal, we find evidence of systematic signal in the process of differentiation of behavioural and emotional problems in early childhood, independently of co‐occurring (i.e., total) problems. It is important to note that the total level of behavioural and emotional problems was a considerably stronger predictor of symptoms in middle childhood than differentiation between these domains. This is consistent with the power of the “p” factor in predicting future outcomes, which is well established (Caspi et al., 2014; Lahey et al., 2015). Nonetheless, the proportion of the variance in middle childhood symptoms accounted for by differentiation was also substantial. Modelling differentiation may provide additional information about the aetiology of specific patterns of emerging mental health problems. Separating general and specific aspects of mental health outcomes as we do here, or by means of a p‐factor model with specific, orthogonal sub‐factors (e.g., Chen et al., 2022) may enhance the informativeness and discriminatory power of any identified risk factors.

Having established that differentiation operationalised as the difference between behavioural and emotional problem scores has predictive validity for later outcomes, we estimated associations with a range of early life exposures. Observational associations were found for maternal at‐risk drinking and parental relationship problems predicting differentiation towards behavioural problems, and maternal prenatal distress, concurrent distress, and parental education predicting differentiation towards emotional problems. However, when adjusting for unobserved familial confounding using a sibling comparison design, most associations were attenuated. First, it is worth noting that random measurement error could be sufficient to attenuate the within‐sibship estimates in comparison to unadjusted estimates, even if there was no confounding (Frisell et al., 2012). Nonetheless, the relative consistency of effect estimates for maternal at‐risk drinking and prenatal distress across observational, unadjusted (sibling only), and adjusted (sibling only) analyses suggests a robustness to these results. Some associations switched sign or seemingly *emerged* after adjustment, which could indicate that familial confounding may, in some cases, mask rather than inflate effect estimates. For instance, paternal prenatal distress seemed to emerge as a predictor of differentiation toward behavioural problems. However, we refrain from ‐ and caution against ‐ interpreting these associations as such. This is partly because they could be unreliable, or biased (to a larger extent than the unadjusted estimate) by confounders not shared by siblings (Frisell et al., 2012). Moreover, despite our attempts to adjust for this, they could be affected by selection bias due to being a sibling participating in MoBa. Due to these alternative possible explanations, we restrict our interpretations to effects that are consistent across the observational, unadjusted, and sibling adjusted analyses, in line with a triangulation approach (Lawlor et al., 2016).

For some early life exposures (e.g., maternal concurrent distress), results were inconsistent across the observational analyses in the whole sample and the unadjusted analyses in the sibling sub‐sample. In this sub‐sample, some selection effects remained after weighting (see Appendix S7 and Figure S6). Selection bias related to repeated participation (i.e., being a sibling vs. a singleton) may be the reason behind these seemingly conflicting results. Also, the associations between early life exposures and the rate of differentiation were of much smaller magnitude in the weighted sibling sub‐sample. This could perhaps suggest that the slope factor picked up effects in the whole sample that were in part due to selective attrition, which were attenuated in the sibling‐only analyses.

The attenuation of the effects of parental education and maternal concurrent distress after accounting for familial confounding is also notable. Parental education is virtually invariant between siblings, which most likely accounts for this. Results for parental education should be considered tentative and warranting further investigation (Costello et al., 2003; Torvik et al., 2020). Similarly, for other risk factors that rarely vary within sibling pairs (i.e., maternal smoking during pregnancy; see within and between cluster variation for each risk factor in Table S8) the adjusted results rely on fewer observations and should not be considered conclusive. Other designs, such as the children‐of‐siblings design (Kuja‐Halkola et al., 2014) are likely superior when estimating the effect of exposures that seldom vary within families on offspring outcomes. The lack of consistent effects of maternal concurrent distress in our study seemingly conflicts with prior reports (i.e., Gjerde et al., 2017). This could be explained by methodological differences between our and previous studies.

### Limitations

There are notable strengths to this study, including the large sample size, the detailed preregistration, and the ability to adjust for confounding by unobserved familial risk. Nevertheless, we acknowledge some limitations of our approach. First, we use a differentiation score and draw a contrast with approaches that combine emotional and behavioural problems into a single, total score. However, it is equally common that these subdomains are investigated independently (as per the sensitivity analyses reported in Figures S4 and S5) ‐ in which case, the information contained is exactly equivalent to a model with both a differentiation and total score. Therefore, it should be noted as a clarification (if not a limitation per se), that using a differentiation score is a way to re‐frame existing information, rather than a way to access novel information from a measure. For our research questions, this re‐framing is pragmatic and helpful; in other cases, use of the variables as separate subdomains may be more appropriate. Second, as with all difference scores, measurement unreliability means that our differentiation scores include noise in addition to signal (Edwards, 1994; see also reliability estimates in Table S1). Here, latent growth factors capture signal that is shared across waves without measurement error, which helps with mitigating this issue. Moreover, our validation results indicate that the differentiation measure does capture meaningful signal. Third, since mothers reported on both the predictors and outcomes, shared method variance might inflate some of the observed associations (Podsakoff et al., 2003). This limitation is inherent to MoBa and similar cohorts, although recent work suggests that any resulting bias may be limited (Olino et al., 2021). Here, any time‐invariant maternal rating bias would to some extent be adjusted for in the multilevel SEM models. In addition we have data on predictors from the fathers, which would be less impacted by shared method variance. Fourth, self‐report of certain risk factors (e.g., smoking during pregnancy) is limited to the extent that parents are willing to report on such sensitive topics. Fifth, differentiation could result from unreliability of symptom measures and clinician‐assigned diagnoses. However, this would again be adjusted for in the multilevel SEM. Finally, we found some evidence of selective attrition in our sample, although this was of limited scope to bias the results. Overall, MoBa is not fully representative of the general population due to non‐participation bias. Previous studies have found that the youngest women, those living alone, smokers, women with previous stillbirths, and women with more than two previous births, are under‐represented in MoBa (Nilsen et al., 2009). However, sensitivity analyses using inverse probability of participation weighting to account for potential biases showed some, but overall limited, differences between weighted and unweighted results (see Figures S4 and S5 for the validation models, and Tables S13‐S20 for the predictor models).

## CONCLUSION

We showed that differentiation of behavioural and emotional problems in early childhood was associated with symptoms of mental health conditions in middle childhood, and clinical manifestations of these conditions later in childhood and adolescence. These effects were independent of the total extent of individuals' behavioural and emotional problems in early childhood. Further, we identified specific predictors of differentiation remaining after adjustment for confounding by unobserved familial risk. Identifying modifiable factors associated with differentiation of behavioural and emotional problems in early life may help efforts to detect and eventually prevent impairment from mental health conditions.

## AUTHOR CONTRIBUTIONS

**Adrian Dahl Askelund**: Conceptualization; Data curation; Formal analysis; Methodology; Project administration; Validation; Visualization; Writing – original draft; Writing – review & editing. **Helga Ask**: Funding acquisition; Writing – review & editing. **Eivind Ystrom**: Conceptualization; Writing – review & editing. **Alexandra Havdahl**: Conceptualization; Funding acquisition; Methodology; Resources; Supervision; Writing – review & editing. **Laurie J. Hannigan**: Conceptualization; Data curation; Formal analysis; Funding acquisition; Methodology; Software; Supervision; Validation; Visualization; Writing – original draft; Writing – review & editing.

## CONFLICT OF INTEREST STATEMENT

Eivind Ystrom is a Joint Editor for JCPP Advances. The remaining authors have declared that they have no competing or potential conflicts on interest.

### OPEN RESEARCH BADGES

This article has been awarded Open Materials and Preregistered Research Designs badges. Data preparation and analysis code is publicly accessible via Github at https://github.com/psychgen/childhood‐differentiation. Learn more about the Open Practices badges from the Center for Open Science: https://osf.io/s2wqx/.

## Ethical considerations

Written informed consent was obtained from all participants upon recruitment. The initial data collection was based on a licence from the Norwegian Data Protection Agency and approval from The Regional Committee for Medical Research Ethics. The MoBa cohort is currently regulated by the Norwegian Health Registry Act. Ethical approval for this work has been given by The Regional Committees for Medical and Health Research Ethics (2016/1702).

## Supporting information

Supplementary Information S1

## Data Availability

The MoBa data are not publicly available as the consent given by the participants does not open for storage of data on an individual level in repositories or journals. Researchers who want access to data sets for replication should submit an application to datatilgang(at)fhi.no. Access to datasets requires approval from The Regional Committee for Medical and Health Research Ethics in Norway and an agreement with MoBa. Data preparation and analysis code for all elements of the project is publicly available on Github: https://github.com/psychgen/childhood‐differentiation.

## References

[jcv212176-bib-0001] Allegrini, A. G. , Cheesman, R. , Rimfeld, K. , Selzam, S. , Pingault, J.‐B. , Eley, T. C. , & Plomin, R. (2020). The p factor: Genetic analyses support a general dimension of psychopathology in childhood and adolescence. Journal of Child Psychology and Psychiatry, 61(1), 30–39. 10.1111/jcpp.13113 31541466 PMC6906245

[jcv212176-bib-0002] Angold, A. , Costello, E. J. , Messer, S. C. , & Pickles, A. (1995). Development of a short questionnaire for use in epidemiological studies of depression in children and adolescents. International Journal of Methods in Psychiatric Research, 5, 237–249.

[jcv212176-bib-0003] Auersperg, F. , Vlasak, T. , Ponocny, I. , & Barth, A. (2019). Long‐term effects of parental divorce on mental health–A meta‐analysis. Journal of Psychiatric Research, 119, 107–115. 10.1016/j.jpsychires.2019.09.011 31622869

[jcv212176-bib-0004] Benjamini, Y. , & Hochberg, Y. (1995). Controlling the False Discovery rate: A practical and powerful approach to multiple testing. Journal of the Royal Statistical Society: Series B, 57(1), 289–300. 10.1111/j.2517-6161.1995.tb02031.x

[jcv212176-bib-0005] Birmaher, B. , Khetarpal, S. , Brent, D. , Cully, M. , Balach, L. , Kaufman, J. , & Neer, S. M. (1997). The screen for child anxiety related emotional disorders (SCARED): Scale construction and psychometric characteristics. Journal of the American Academy of Child & Adolescent Psychiatry, 36(4), 545–553. 10.1097/00004583-199704000-00018 9100430

[jcv212176-bib-0006] Brion, M.‐J. , Victora, C. , Matijasevich, A. , Horta, B. , Anselmi, L. , Steer, C. , Menezes, A. M. B. , Lawlor, D. A. , & Davey Smith, G. (2010). Maternal smoking and child psychological problems: Disentangling causal and noncausal effects. Pediatrics, 126(1), e57–e65. 10.1542/peds.2009-2754 20587678 PMC3605780

[jcv212176-bib-0007] Caspi, A. , Houts, R. M. , Belsky, D. W. , Goldman‐Mellor, S. J. , Harrington, H. , Israel, S. , Meier, M. H. , Ramrakha, S. , Shalev, I. , Moffitt, T. E. , & Poulton, R. (2014). The p factor: One general psychopathology factor in the structure of psychiatric disorders? Clinical Psychological Science, 2(2), 119–137. 10.1177/2167702613497473 25360393 PMC4209412

[jcv212176-bib-0008] Caspi, A. , Houts, R. M. , Belsky, D. W. , Harrington, H. , Hogan, S. , Ramrakha, S. , Poulton, R. , & Moffitt, T. E. (2016). Childhood forecasting of a small segment of the population with large economic burden. Nature Human Behaviour, 1(1), 1–10. 10.1038/s41562-016-0005 PMC5505663 28706997

[jcv212176-bib-0009] Caspi, A. , Moffitt, T. E. , Newman, D. L. , & Silva, P. A. (1996). Behavioral observations at age 3 years predict adult psychiatric disorders. Longitudinal evidence from a birth cohort. Archives of General Psychiatry, 53(11), 1033–1039. 10.1001/archpsyc.1996.01830110071009 8911226

[jcv212176-bib-0010] Cheesman, R. , Eilertsen, E. M. , Ahmadzadeh, Y. I. , Gjerde, L. C. , Hannigan, L. J. , Havdahl, A. , Young, A. I. , Eley, T. C. , Njølstad, P. R. , Andreassen, O. A. , Ystrom, E. , McAdams, T. A. , & Magnus, P. (2020). How important are parents in the development of child anxiety and depression? A genomic analysis of parent‐offspring trios in the Norwegian mother father and child cohort study (MoBa). BMC Medicine, 18(1), 1–11. 10.1186/s12916-020-01760-1 33106172 PMC7590735

[jcv212176-bib-0011] Chen, C. , Lu, Y. , Lundström, S. , Larsson, H. , Lichtenstein, P. , & Pettersson, E. (2022). Associations between psychiatric polygenic risk scores and general and specific psychopathology symptoms in childhood and adolescence between and within dizygotic twin pairs. Journal of Child Psychology and Psychiatry, 63(12), 1513–1522. 10.1111/jcpp.13605 35292971 PMC9790278

[jcv212176-bib-0012] Costello, E. J. , Compton, S. N. , Keeler, G. , & Angold, A. (2003). Relationships between poverty and psychopathology: A natural experiment. JAMA, 290(15), 2023–2029. 10.1001/jama.290.15.2023 14559956

[jcv212176-bib-0013] Dearing, E. , McCartney, K. , & Taylor, B. A. (2006). Within‐child associations between family income and externalizing and internalizing problems. Developmental Psychology, 42(2), 237–252. 10.1037/0012-1649.42.2.237 16569163

[jcv212176-bib-0014] Edwards, J. R. (1994). Regression analysis as an alternative to difference scores. Journal of Management, 20(3), 683–689. 10.1177/014920639402000311

[jcv212176-bib-0015] Eilertsen, E. M. , Cheesman, R. , Ayorech, Z. , Røysamb, E. , Pingault, J.‐B. , Njølstad, P. R. , Andreassen, O. A. , Havdahl, A. , McAdams, T. A. , Ystrøm, E. , & Torvik, F. A. (2022). On the importance of parenting in externalizing disorders: An evaluation of indirect genetic effects in families. Journal of Child Psychology and Psychiatry, 63(10), 1186–1195. 10.1111/jcpp.13654 35778910 PMC9796091

[jcv212176-bib-0016] Enders, C. K. , & Bandalos, D. L. (2001). The relative performance of full information maximum likelihood estimation for missing data in structural equation models. Structural Equation Modeling: A Multidisciplinary Journal, 8(3), 430–457. 10.1207/s15328007sem0803_5

[jcv212176-bib-0017] Frisell, T. (2021). Invited commentary: Sibling‐comparison designs, are they worth the effort? American Journal of Epidemiology, 190(5), 738–741. 10.1093/aje/kwaa183 32830847 PMC8096426

[jcv212176-bib-0018] Frisell, T. , Öberg, S. , Kuja‐Halkola, R. , & Sjölander, A. (2012). Sibling comparison designs: Bias from non‐shared confounders and measurement error. Epidemiology, 23(5), 713–720. 10.1097/ede.0b013e31825fa230 22781362

[jcv212176-bib-0019] Gjerde, L. C. , Eilertsen, E. M. , Hannigan, L. J. , Eley, T. , Røysamb, E. , Reichborn‐Kjennerud, T. , Rijsdijk, F. V. , McAdams, T. A. , & Ystrom, E. (2021). Associations between maternal depressive symptoms and risk for offspring early‐life psychopathology: The role of genetic and non‐genetic mechanisms. Psychological Medicine, 51(3), 441–449. 10.1017/s0033291719003301 31813389

[jcv212176-bib-0020] Gjerde, L. C. , Eilertsen, E. M. , Reichborn‐Kjennerud, T. , McAdams, T. A. , Zachrisson, H. D. , Zambrana, I. M. , Røysamb, E. , Kendler, K. S. , & Ystrom, E. (2017). Maternal perinatal and concurrent depressive symptoms and child behavior problems: A sibling comparison study. Journal of Child Psychology and Psychiatry, 58(7), 779–786. 10.1111/jcpp.12704 28229455 PMC5484352

[jcv212176-bib-0021] Goodman, S. H. , Rouse, M. H. , Connell, A. M. , Broth, M. R. , Hall, C. M. , & Heyward, D. (2011). Maternal depression and child psychopathology: A meta‐analytic review. Clinical Child and Family Psychology Review, 14(1), 1–27. 10.1007/s10567-010-0080-1 21052833

[jcv212176-bib-0022] Hallquist, M. N. , & Wiley, J. F. (2018). MplusAutomation: An R package for facilitating large‐scale latent variable analyses in M plus. Structural Equation Modeling: A Multidisciplinary Journal, 25(4), 621–638. 10.1080/10705511.2017.1402334 30083048 PMC6075832

[jcv212176-bib-0023] Hannigan, L. J. , Walaker, N. , Waszczuk, M. A. , McAdams, T. A. , & Eley, T. C. (2017). Aetiological influences on stability and change in emotional and behavioural problems across development: A systematic review. Psychopathology Review, 4(1), 52–108. 10.5127/pr.038315 28337341 PMC5360234

[jcv212176-bib-0024] Jami, E. S. , Hammerschlag, A. R. , Bartels, M. , & Middeldorp, C. M. (2021). Parental characteristics and offspring mental health and related outcomes: A systematic review of genetically informative literature. Translational Psychiatry, 11(1), 197. 10.1038/s41398-021-01300-2 33795643 PMC8016911

[jcv212176-bib-0025] Jenkins, J. M. , & Smith, M. A. (1991). Marital disharmony and children’s behaviour problems: Aspects of a poor marriage that affect children adversely. Journal of Child Psychology and Psychiatry, 32(5), 793–810. 10.1111/j.1469-7610.1991.tb01903.x 1918229

[jcv212176-bib-0026] Kendler, K. S. , Abrahamsson, L. , Ohlsson, H. , Sundquist, J. , & Sundquist, K. (2022). An extended Swedish adoption study of anxiety disorder and its cross‐generational familial relationship with major depression. American Journal of Psychiatry, 179(9), 640–649. 10.1176/appi.ajp.21111110 36048482

[jcv212176-bib-0027] Kendler, K. S. , & Baker, J. H. (2007). Genetic influences on measures of the environment: A systematic review. Psychological Medicine, 37(05), 615–626. 10.1017/s0033291706009524 17176502

[jcv212176-bib-0028] Kline, R. B. (2011). Principles and practice of structural equation modeling (3rd ed.). Guilford.

[jcv212176-bib-0029] Kuja‐Halkola, R. , D’Onofrio, B. M. , Larsson, H. , & Lichtenstein, P. (2014). Maternal smoking during pregnancy and adverse outcomes in offspring: Genetic and environmental sources of covariance. Behavior Genetics, 44(5), 456–467. 10.1007/s10519-014-9668-4 25117564 PMC4194213

[jcv212176-bib-0030] Lahey, B. B. , Rathouz, P. J. , Keenan, K. , Stepp, S. D. , Loeber, R. , & Hipwell, A. E. (2015). Criterion validity of the general factor of psychopathology in a prospective study of girls. Journal of Child Psychology and Psychiatry, 56(4), 415–422. 10.1111/jcpp.12300 25052460 PMC4435546

[jcv212176-bib-0031] Lawlor, D. A. , Tilling, K. , & Davey Smith, G. (2016). Triangulation in aetiological epidemiology. International Journal of Epidemiology, 45(6), 1866–1886. 28108528 10.1093/ije/dyw314 PMC5841843

[jcv212176-bib-0032] Leventhal, T. , & Dupéré, V. (2019). Neighborhood effects on children’s development in experimental and nonexperimental research. Annual Review of Developmental Psychology, 1, 149–176. 10.1146/annurev-devpsych-121318-085221

[jcv212176-bib-0033] Liming, K. W. , & Grube, W. A. (2018). Wellbeing outcomes for children exposed to multiple adverse experiences in early childhood: A systematic review. Child and Adolescent Social Work Journal, 35(4), 317–335. 10.1007/s10560-018-0532-x

[jcv212176-bib-0034] Lund, I. O. , Eilertsen, E. M. , Gjerde, L. C. , Torvik, F. A. , Røysamb, E. , Reichborn‐Kjennerud, T. , & Ystrom, E. (2020). Maternal drinking and child emotional and behavior problems. Pediatrics, 145(3), e20192007. 10.1542/peds.2019-2007 PMC7053080 32094288

[jcv212176-bib-0035] Magnus, P. , Birke, C. , Vejrup, K. , Haugan, A. , Alsaker, E. , Daltveit, A. K. , Handal, M. , Haugen, M. , Høiseth, G. , Knudsen, G. P. , Paltiel, L. , Schreuder, P. , Tambs, K. , Vold, L. , & Stoltenberg, C. (2016). Cohort profile update: The Norwegian mother and child cohort study (MoBa). International Journal of Epidemiology, 45(2), 382–388. 10.1093/ije/dyw029 27063603

[jcv212176-bib-0036] Magnus, P. , Irgens, L. M. , Haug, K. , Nystad, W. , Skjærven, R. , & Stoltenberg, C. (2006). Cohort profile: The Norwegian mother and child cohort study (MoBa). International Journal of Epidemiology, 35(5), 1146–1150. 10.1093/ije/dyl170 16926217

[jcv212176-bib-0037] Mulraney, M. , Coghill, D. , Bishop, C. , Mehmed, Y. , Sciberras, E. , Sawyer, M. , Efron, D. , & Hiscock, H. (2021). A systematic review of the persistence of childhood mental health problems into adulthood. Neuroscience & Biobehavioral Reviews, 129, 182–205. 10.1016/j.neubiorev.2021.07.030 34363845

[jcv212176-bib-0038] Murray, A. L. , Eisner, M. , & Ribeaud, D. (2016). The development of the general factor of psychopathology ‘p factor’through childhood and adolescence. Journal of Abnormal Child Psychology, 44(8), 1573–1586. 10.1007/s10802-016-0132-1 26846993

[jcv212176-bib-0039] Muthén, L. K. , & Muthén, B. O. (1998). Mplus user’s guide (8th ed.). Muthén & Muthén. 1998‐2017.

[jcv212176-bib-0040] Nilsen, R. M. , Vollset, S. E. , Gjessing, H. K. , Skjaerven, R. , Melve, K. K. , Schreuder, P. , Alsaker, E. R. , Haug, K. , Daltveit, A. K. , & Magnus, P. (2009). Self‐selection and bias in a large prospective pregnancy cohort in Norway. Paediatric & Perinatal Epidemiology, 23(6), 597–608. 10.1111/j.1365-3016.2009.01062.x 19840297

[jcv212176-bib-0041] Olino, T. M. , Michelini, G. , Mennies, R. J. , Kotov, R. , & Klein, D. N. (2021). Does maternal psychopathology bias reports of offspring symptoms? A study using moderated non‐linear factor analysis. Journal of Child Psychology and Psychiatry, 62(10), 1195–1201. 10.1111/jcpp.13394 33638150

[jcv212176-bib-0042] Plomin, R. , DeFries, J. C. , & Loehlin, J. C. (1977). Genotype‐environment interaction and correlation in the analysis of human behavior. Psychological Bulletin, 84(2), 309–322. 10.1037/0033-2909.84.2.309 557211

[jcv212176-bib-0043] Podsakoff, P. M. , MacKenzie, S. B. , Lee, J.‐Y. , & Podsakoff, N. P. (2003). Common method biases in behavioral research: A critical review of the literature and recommended remedies. Journal of Applied Psychology, 88(5), 879–903. 10.1037/0021-9010.88.5.879 14516251

[jcv212176-bib-0044] Polderman, T. J. , Benyamin, B. , De Leeuw, C. A. , Sullivan, P. F. , Van Bochoven, A. , Visscher, P. M. , & Posthuma, D. (2015). Meta‐analysis of the heritability of human traits based on fifty years of twin studies. Nature Genetics, 47(7), 702–709. 10.1038/ng.3285 25985137

[jcv212176-bib-0045] Rice, F. , Lewis, G. , Harold, G. T. , & Thapar, A. (2013). Examining the role of passive gene–environment correlation in childhood depression using a novel genetically sensitive design. Development and Psychopathology, 25(1), 37–50. 10.1017/s0954579412000880 23398751

[jcv212176-bib-0046] Scott, J. , Martin, G. , Welham, J. , Bor, W. , Najman, J. , O’Callaghan, M. , Williams, G. , Aird, R. , & McGrath, J. (2009). Psychopathology during childhood and adolescence predicts delusional‐like experiences in adults: A 21‐year birth cohort study. American Journal of Psychiatry, 166(5), 567–574. 10.1176/appi.ajp.2008.08081182 19339357

[jcv212176-bib-0047] Silva, R. R. , Alpert, M. , Pouget, E. , Silva, V. , Trosper, S. , Reyes, K. , & Dummit, S. (2005). A rating scale for disruptive behavior disorders, based on the DSM‐IV item pool. Psychiatric Quarterly, 76(4), 327–339. 10.1007/s11126-005-4966-x 16217627

[jcv212176-bib-0048] Sterba, S. K. , Copeland, W. , Egger, H. L. , Jane Costello, E. , Erkanli, A. , & Angold, A. (2010). Longitudinal dimensionality of adolescent psychopathology: Testing the differentiation hypothesis. Journal of Child Psychology and Psychiatry, 51(8), 871–884. 10.1111/j.1469-7610.2010.02234.x 20345843 PMC3630513

[jcv212176-bib-0049] Torvik, F. A. , Eilertsen, E. M. , McAdams, T. A. , Gustavson, K. , Zachrisson, H. D. , Brandlistuen, R. , Gjerde, L. C. , Havdahl, A. , Stoltenberg, C. , Ystrom, E. , & Ask, H. (2020). Mechanisms linking parental educational attainment with child ADHD, depression, and academic problems: A study of extended families in the Norwegian mother, father and child cohort study. Journal of Child Psychology and Psychiatry, 61(9), 1009–1018. 10.1111/jcpp.13197 31957030 PMC8607471

