# Supplementary material for: Exploring the differentiation of behavioural and emotional problems across childhood: A prospective longitudinal cohort study
Source: JCPP Adv. 2023 Jun 30;3(4):e12176. doi: 10.1002/jcv2.12176 (PMC10694541; doi:10.1002/jcv2.12176)
Supplement: Supplementary file 1 — Supplementary Information S1 [file JCV2-3-e12176-s001.docx]

# Supporting Information

###

***Askelund et al. Exploring the differentiation of behavioural and emotional problems across childhood: a prospective longitudinal cohort study***

## Supporting Methods

### Appendix S1: Psychometric properties

When children were aged 1.5 years, 3 years, and 5 years, mothers responded to items on the CBCL using a 3-point scale ranging from “Not true” to “Often true” to describe their children’s behavioural and emotional problems. In our study, the behavioural problems subscale comprised 8 items assessing attention problems and aggressive behaviour. The emotional problems subscale comprised 5 items assessing the extent to which children are emotionally reactive, anxious/depressed, have somatic complaints and are withdrawn. At child age 8, mothers responded to items on the SMFQ and SCARED using a 3-point scale ranging from “Not true” to “True” to describe their children’s symptoms. The response set for the RS-DBD items was “Never/rarely”, “Sometimes”, “Often”, and “Very often” as mothers rated how often their children engaged in different behaviours. Internal consistency for consistent items on the CBCL behavioural and emotional subscales, and difference scores based on the two, are in Table S1. Internal consistency for the SCARED, SMFQ, and RS-DBD subscales at age 8 are in Table S2.

### Appendix S2: Diagnostic codes

We obtained diagnoses of mental health conditions from medical records. KUHR covers primary health care (using codes from The International Classification of Primary Care; ICPC-2), and NPR covers all public specialist health-care services in Norway (using codes from ICD-10). Note that at the time of analysis, KUHR covered the years 2008-2021 whereas NPR covered the years 2008-2018. We extracted information on diagnoses of depression (ICPC-2: P76; ICD-10: F32-F33, F34.1), anxiety (ICPC-2: P74, P79, P82; ICD-10: F40-F44, F93.0-F93.2), ADHD (ICPC-2: P81; ICD-10: F90), and disruptive behaviour disorders (DBD; ICPC-2: P23; ICD-10: F91-F92).

### Appendix S3: Deviations from preregistration

We have made the following deviations from the preregistration (which can be accessed here: [10.17605/OSF.IO/75HZA](https://doi.org/10.17605/OSF.IO/75HZA)), in addition to those reported in the main manuscript.

- S3.1: We deviated from the planned scoring of alcohol use based on frequency and number of units, as the initial model indicated a protective effect of parental alcohol use with regard to child outcomes in the total model, and a positive inter-relationship with indicators of socioeconomic status such as education and income. We instead used the number of units to code at-risk drinking in each parent based on the procedure in Lund et al., 2020, which was negatively associated with education and income. The reported typical unit of alcohol consumption was coded as follows: “1 to 2” = 0, “3 to 4” = 0.5, “5 to 6” = 1, “7 to 9” = 2, and “10” = 3 for weekends and “1 to 2” = 0, “3 to 4” = 1, “5 to 6” = 2, “7 to 9” = 3, and “10” = 4 for weekdays. We used the average of weekends and weekdays. According to their recommendations, abstaining (i.e., < 1 unit) was coded as missing as it is a form of censored data, and we have other auxiliary information in the data.
- S3.2: Due to a problem with the generated age at questionnaire completion variable, we have replaced this with age at questionnaire return.
- S3.3: Due to low reliability, we decided (prior to running models) to drop the Life Time History of Major Depression (LTH MD) retrospective measure of maternal prenatal depression as a predictor. This is likely to be better captured by SCL-5 and SCL-8 measuring anxiety and depressive symptoms concurrently during pregnancy, which were already included in combined form as measures of maternal prenatal distress.
- S3.4: We used the father’s own report of their income during week 17 of pregnancy since the item where mothers reported the father’s income was not available.
- S3.5: Since the data became more readily available during the time of our analyses, we added ICPC-2 diagnoses from KUHR in addition to ICD-10 diagnoses from NPR.
- S3.6: We added parity as a covariate in addition to sex and age, given the theorised role of birth order in differentiation among siblings. Adding this covariate and re-running the models did not change the pattern of results.
- S3.7: To validate our approach, we planned to test the hypothesis that the association between differentiation in early childhood and symptoms in middle childhood would be better explained by domain-specific pathways than via a general psychopathology (“p”) factor. However, the p factor model explaining scale-level symptoms of depression, anxiety, hyperactivity, inattention, CD, and ODD at age 8 with a single factor showed poor model fit in our sample (CFI = 0.91, TLI = 0.85, RMSEA = 0.13, SRMR = 0.05). Therefore, we ran a correlated factor model without the p factor, representing a deviation from our preregistration.
- S3.8: The growth mixture models were omitted from the manuscript, to streamline the analyses and improve the clarity of the approach.
- S3.9: Due to software limitations, we were unable to run the planned multilevel SEM model with both nuclear and extended family levels. Instead, we ran a 2-level model accounting for nuclear-family level confounding.

### Appendix S4: Definition/removal of outliers

We retained all valid responses to the questionnaires. If participants ticked multiple boxes on a single-response item, their response on this item was set to missing. If respondents completed less than half of the items for a given scale, their scale score was not computed and their data for this variable was considered missing.

### Appendix S5: Inverse probability weighting

*Initial participation in MoBa*

To test the influence of potential biases from non-random initial participation in MoBa on the estimated associations, we conducted sensitivity analyses incorporating inverse probability of participation (IPP) weights. We obtained aggregated statistics on parity and cohabitation status from Statistics Norway, and generated weights based on differences between women participating in MoBa and women who gave birth in Norway during the recruitment window, but did not participate. These variables were selected as the basis for the IPP model because they were anticipated to predict participation in MoBa, were in the analytic sample used for analyses, and were included in a publicly available summary dataset of live births during MoBa recruitment years. The logistic regression model to generate IPP weights used only data from the years 2003-2008 - the years in which aggregate data was available that covered the main MoBa recruitment period - though all individuals in MoBa were ultimately able to be assigned a weight on the basis of their observed values for parity and cohabitation status. The model correctly predicted participation status in 77% of cases among women giving birth between 2003-2008, and at 73% overall. Stabilised IPP weights were generated using the ipw R package (version 1.2; van der Wal & Geskus, 2011), meaning that the marginal probability of participation, rather than 1, was used as the numerator in the calculation of weights.

*Repeated (i.e., sibling) participation in MoBa*

A potential source of bias in our analyses using only sibling data might be *repeated* participation. To the extent that families who participate in MoBa with more than one child differ from those participating only once on relevant variables, results are likely to be biased relative to the overall MoBa sample (over and above any differences compared to the general population). To adjust our models using only sibling data for biases from repeated participation in MoBa (i.e., being a sibling vs a singleton), we include inverse probability weights derived from a model regressing repeated participation on all other variables in the dataset. These weights were derived in a similar fashion to the IPP weights, described above, with one important difference. To avoid listwise deletion for missing data, we first multiply imputed 60 datasets with 15 iterations each using the *mice R* package (version 3.13.0; van Buuren & Groothuis-Oudshoorn, 2011). Smoothed, stabilised weights were generated by averaging across stabilised weights from all 60 datasets. Models predicted sibling status successfully, on average, 69% of the time.

R code used to generate the inverse probability of initial and repeated participation weights is available, with the other analytic code from the project, on Github: https://github.com/psychgen/childhood-differentiation.

References:

van Buuren, S., & Groothuis-Oudshoorn, K. (2011). mice: Multivariate imputation by chained equations in R. *Journal of Statistical Software*, *45*(3), 1-67.

van der Wal, W. M., & Geskus, R. B. (2011). ipw: an R package for inverse probability weighting. *Journal of Statistical Software*, *43*(13), 1-23.

## Supporting Results

### Appendix S6: Selective attrition

There was evidence of some selective attrition based on the CBCL subscales, where those who were missing at 5 years scored slightly higher at 1.5 years for behavioural problems (t_75713_ = -11.02, mean = 3.85 with data vs 4.03 without data, diff = -0.18 [-0.21, -0.15]) and emotional problems (t_69356_ = -6.94, mean = 1.30 with data vs 1.36 without data, diff = -0.06 [-0.08, -0.05]).

***Appendix S7: Sibling weights***

Differences between siblings and singletons in most early life exposures were attenuated after employing the sibling weights, with the exception of paternal at-risk drinking and relationship problems (see Figure S6).

## Supporting Tables

| **Variable** | **Age** | **Ordinal alpha** |
| --- | --- | --- |
| Behavioural problems | 1.5yr | 0.70 |
|  | 3yr | 0.77 |
|  | 5yr | 0.81 |
| Emotional problems | 1.5yr | 0.66 |
|  | 3yr | 0.68 |
|  | 5yr | 0.73 |
| Differentiation  (behavioural - emotional) | 1.5yr | 0.58 |
|  | 3yr | 0.58 |
|  | 5yr | 0.65 |

**Table S1. Ordinal alphas for Child Behavior Checklist (CBCL) subscales,
and the difference scores based on these subscales at each time point.**

| **Variable** | **Ordinal alpha** |
| --- | --- |
| Hyperactivity | 0.91 |
| Inattention | 0.92 |
| Conduct disorder | 0.88 |
| Oppositional defiant disorder | 0.91 |
| Anxiety | 0.76 |
| Depression | 0.92 |

**Table S2. Ordinal Cronbach’s alphas for symptom scales measured at 8 years.**

| **Variable** | **Role** | **Measure** | **Items** | **Processing** |
| --- | --- | --- | --- | --- |
| Behavioural/emotional  problems differentiation | Growth variable | CBCL | 15 | beh & emo scale scores calculated as mean (available_items)* total_items (IF available_items > total_items/2) at age 1.5y,  3y & 5y; standardised mean 0, SD 1; differentiation = behavioural - emotional; total = behavioural + emotional |
| Parental income | Predictor | Single item | 2 | income score calculated as mean of maternal and paternal gross income at week 17 of pregnancy |
| Parental education | Predictor | Single item | 2 | education score calculated as mean of maternal and paternal education completed at week 17 of pregnancy |
| Maternal prenatal life events | Predictor | Life events | 8 | pre_stress_m score calculated as mean (available items)*total items (IF available_items > total_items/2) at week 30 of pregnancy |
| Maternal life events | Predictor | Life events | 10 | post_stress_m score calculated as mean (available items)*total items (IF available_items > total_items/2) of age 6m + 1.5y |
| Paternal life events | Predictor | Life events | 11 | stress_f score calculated as mean (available items)*total items (IF available_items > total_items/2) at week 17 of pregnancy |
| Maternal relationship problems | Predictor | RSS | 10 | relation_m score calculated as mean (available items)*total items (IF available_items > total_items/2)*-1 at age 1.5y |
| Paternal relationship problems | Predictor | RSS | 5 | relation_f score calculated as mean (available items)*total items (IF available_items > total_items/2)*-1 at week 17 of pregnancy |
| Maternal prenatal smoking | Predictor | Single item | 1 | smoking_m score calculated as mean of week 17 + 30 of pregnancy |
| Paternal prenatal smoking | Predictor | Single item | 1 | smoking_f score based on item at week 17 of pregnancy |
| Maternal at-risk drinking | Predictor | Single item | 2 | alc_risk_tot_m score calculated as mean (number of units weekends + weekdays/2) at age 1.5y + 3y |
| Paternal at-risk drinking | Predictor | Single item | 2 | alc_risk_tot_f score calculated as mean (number of units weekends + weekdays/2) at week 17 of pregnancy |
| Maternal alcohol problems | Predictor | RAPI | 5 | alc_prob_m score calculated as mean (available items)*total items (IF available_items > total_items/2) at week 17 of pregnancy |
| Maternal prenatal distress | Predictor | SCL-5 | 5 | pre_distress_m score calculated as mean (available items)*total items (IF available_items > total_items/2) of w17+30 of pregnancy |
| Paternal prenatal distress | Predictor | SCL-8 | 8 | pre_distress_f score calculated as mean (available items)*total items (IF available_items > total_items/2) at week 17 of pregnancy |
| Maternal distress | Predictor | SCL-8 | 8 | distress_m score calculated as mean (available items)*total items (IF available_items > total_items/2) of age 1.5y + 3y |
| Maternal postnatal depression | Predictor | EPDS | 6 | post_dep_m score calculated as mean (available items)*total items (IF available_items > total_items/2) at age 6m |
| Depression symptoms | Outcome | SMFQ | 13 | dep_c score calculated as mean (available items)*total items (IF available_items > total_items/2) at age 8y |
| Anxiety symptoms | Outcome | SCARED | 5 | anx_c score calculated as mean (available items)*total items (IF available_items > total_items/2) at age 8y |
| Hyperactivity symptoms | Outcome | RS-DBD | 9 | hyp_c score calculated as mean (available items)*total items (IF available_items > total_items/2) at age 8y |
| Inattention symptoms | Outcome | RS-DBD | 9 | inat_c score calculated as mean (available items)*total items (IF available_items > total_items/2) at age 8y |
| Conduct disorder symptoms | Outcome | RS-DBD | 8 | cd_c score calculated as mean (available items)*total items (IF available_items > total_items/2) at age 8y |
| Oppositional defiant symptoms | Outcome | RS-DBD | 8 | odd_c score calculated as mean (available items)*total items (IF available_items > total_items/2) at age 8y |
| Psychiatric diagnoses | Outcome | Registry |  | diagnoses from ICPC-2 (P76; P74; P79; P82; P81; P23) and/or ICD-10 (F32-F33; F34.1; F40-F44; F93.0-F93.2; F90; F91-F92) |
| Child sex | Covariate | Registry |  | sex registered at birth dummy coded as: males = 0, females = 1 |
| Parity | Covariate | Registry |  | number of previous deliveries registered, categorised from 0-4 |
| Child age | Covariate | Single item |  | child age when the questionnaire was returned |

**Table S3. Overview of variables included in the study, with information about their role, and how they are measured and processed.***Note: beh = behavioural problems; emo = emotional problems; m = mother; f = father; c = child; CBCL = Child Behaviour Checklist; RSS = Relationship Satisfaction Scale; RAPI = Rutgers Alcohol Problems Index; SCL-8 = The (Hopkins) Symptom Checklist; EPDS = Edinburgh Postnatal Depression Scale; SMFQ = Short Mood and Feelings Questionnaire; SCARED = Screen for Child Anxiety and Related Disorders; RS-DBD = Parent/Teacher Rating Scale for Disruptive Behaviour Disorder.*

##

| **Measure** | **Age** | **N** | **Mean** | **SD** | **Min** | **Max** |  |
| --- | --- | --- | --- | --- | --- | --- | --- |
| Emotional problems (CBCL) | 1.5 | 70726 | 1.33 | 1.24 | 0 | 10 |  |
| Behavioural problems (CBCL) | 1.5 | 75694 | 3.94 | 2.27 | 0 | 16 |  |
| Emotional problems (CBCL) | 3 | 58165 | 1.41 | 1.41 | 0 | 10 |  |
| Behavioural problems (CBCL) | 3 | 58171 | 3.87 | 2.43 | 0 | 16 |  |
| Emotional problems (CBCL) | 5 | 41218 | 1.08 | 1.31 | 0 | 10 |  |
| Behavioural problems (CBCL) | 5 | 41218 | 2.50 | 2.28 | 0 | 16 |  |
| Depressive symptoms (SMFQ) | 8 | 43247 | 1.87 | 2.45 | 0 | 26 |  |
| Anxiety symptoms (SCARED) | 8 | 43331 | 1.03 | 1.20 | 0 | 10 |  |
| Inattention symptoms (RS-DBD) | 8 | 43298 | 4.98 | 4.14 | 0 | 27 |  |
| Hyperactivity symptoms (RS-DBD) | 8 | 43291 | 3.56 | 3.91 | 0 | 27 |  |
| Conduct symptoms (RS-DBD) | 8 | 43333 | 0.78 | 1.51 | 0 | 16 |  |
| Oppositional defiant symptoms (RS-DBD) | 8 | 43288 | 3.42 | 3.16 | 0 | 24 |  |

**Table S4. Descriptive statistics for all included symptom measures by child age.**

*Note: CBCL = Child Behavior Checklist; SMFQ = Short Mood and Feelings Questionnaire; SCARED =
Screen for Child Anxiety Related Disorders; RS-DBD = Rating Scale for Disruptive Behaviour Disorders.*

##

##

| **CBCL var** | **comparison model** | **df** | **AIC** | **Chisq.diff** | **p(>Chisq)** | **best-fitting model** |
| --- | --- | --- | --- | --- | --- | --- |
| diff | intercept_and_slope | 10 | 1526266 | NA | NA | NA |
| diff | intercept_only | 15 | 1526943 | 633.76 | <0.001 | intercept_and_slope |
| tot | intercept_and_slope | 10 | 1513744 | NA | NA | NA |
| tot | intercept_only | 15 | 1514529 | 694.82 | <0.001 | intercept_and_slope |

**Table S5. Fit statistics from all model comparisons of basic latent growth models.**
*Note: CBCL var = Child Behavior Checklist variable; diff = differentiation; tot = total; df = degrees
of freedom; AIC = Akaike Information Criterion; Chisq.diff = scaled chi-squared difference test.*

##

| **CBCL var** | **comparison model** | **df** | **AIC** | **Chisq.diff** | **p(>Chisq)** | **best-fitting model** |
| --- | --- | --- | --- | --- | --- | --- |
| diff | intercept_and_slope | 34 | 2158450 | NA | NA | NA |
| diff | intercept_only | 40 | 2158498 | 5.44255 | <0.001 | intercept_and_slope |
| diff | slope_only | 40 | 2159012 | 527.425 | <0.001 | intercept_and_slope |
| diff | no_effects | 46 | 2163571 | 4073.098 | <0.001 | intercept_and_slope |
| tot | intercept_and_slope | 34 | 2139001 | NA | NA | NA |
| tot | intercept_only | 40 | 2139042 | 43.268 | <0.001 | intercept_and_slope |
| tot | slope_only | 40 | 2139695 | 579.980 | <0.001 | intercept_and_slope |
| tot | no_effects | 46 | 2151049 | 8968.732 | <0.001 | intercept_and_slope |

**Table S6. Fit statistics from all model comparisons in the validation step of the LGMs.***Note: CBCL var = Child Behavior Checklist variable; diff = differentiation; tot = total; df = degrees of
freedom; AIC = Akaike Information Criterion; Chisq.diff = scaled chi-squared difference test.*

##

##

| **CBCL var** | **comparison model** | **df** | **AIC** | **Chisq.diff** | **p(>Chisq)** | **best-fitting model** |
| --- | --- | --- | --- | --- | --- | --- |
| diff | intercept_and_slope | 20 | 3672711 | NA | NA | NA |
| diff | intercept_only | 38 | 3672819 | 131.06 | <0.001 | intercept_and_slope |
| diff | slope_only | 38 | 3673257 | 528.22 | <0.001 | intercept_and_slope |
| tot | intercept_and_slope | 73 | 4389810 | NA | NA | NA |
| tot | intercept_only | 92 | 4390004 | 241.64 | <0.001 | intercept_and_slope |
| tot | slope_only | 92 | 4394745 | 5100.6 | <0.001 | intercept_and_slope |

**Table S7. Fit statistics from all model comparisons of the LGMs with all predictors.**
*Note: CBCL var = Child Behavior Checklist variable; diff = differentiation; tot = total; df = degrees
of freedom; AIC = Akaike Information Criterion; Chisq.diff = scaled chi-squared difference test.*

| **Predictor** | **Within estimate** | **Within SE** | **Between estimate** | **Between SE** |
| --- | --- | --- | --- | --- |
| Parental income | 0.38 | 0.005 | 0.63 | 0.007 |
| Parental education | 0.09 | 0.001 | 0.93 | 0.006 |
| Maternal prenatal life events | 0.57 | 0.008 | 0.44 | 0.009 |
| Maternal life events | 0.61 | 0.009 | 0.40 | 0.010 |
| Paternal life events | 0.57 | 0.012 | 0.44 | 0.013 |
| Maternal relationship problems | 0.38 | 0.006 | 0.64 | 0.008 |
| Paternal relationship problems | 0.60 | 0.009 | 0.40 | 0.010 |
| Maternal prenatal smoking | 0.14 | 0.002 | 0.92 | 0.006 |
| Paternal prenatal smoking | 0.22 | 0.003 | 0.80 | 0.006 |
| Maternal at-risk drinking | 0.28 | 0.005 | 0.73 | 0.007 |
| Paternal at-risk drinking | 0.32 | 0.014 | 0.68 | 0.017 |
| Maternal alcohol problems | 0.48 | 0.008 | 0.55 | 0.009 |
| Maternal prenatal distress | 0.35 | 0.005 | 0.68 | 0.007 |
| Paternal prenatal distress | 0.46 | 0.007 | 0.55 | 0.009 |
| Maternal distress | 0.33 | 0.005 | 0.69 | 0.007 |
| Maternal postnatal depression | 0.46 | 0.007 | 0.54 | 0.008 |

**Table S8. Variation within and between sibling pairs for each predictor in the multilevel model.**
*Note: Estimates with corresponding standard errors (SE) of the variation within and between sibling pairs, to
ascertain which of the included predictors have meaningful within-level variance.*

| **Table S9: Sensitivity analysis with behavioural problems as the outcome (unadjusted)** | | | | | | | | |
| --- | --- | --- | --- | --- | --- | --- | --- | --- |
|  | **Intercept** | | | | **Slope** | | | |
| **Predictor** | **EST** | **LCI** | **UCI** | **P_FDR_** | **EST** | **LCI** | **UCI** | **P_FDR_** |
| Paternal prenatal distress | 0.00 | -0.03 | 0.02 | 0.98 | 0.00 | -0.01 | 0.01 | 0.92 |
| Maternal alcohol problems | 0.05 | -0.01 | 0.12 | 0.24 | 0.01 | -0.01 | 0.03 | 0.92 |
| Maternal at-risk drinking | 0.03 | -0.14 | 0.20 | 0.95 | 0.00 | -0.06 | 0.05 | 0.92 |
| Paternal at-risk drinking | -0.01 | -0.66 | 0.64 | 0.98 | -0.01 | -0.21 | 0.19 | 0.92 |
| Paternal prenatal smoking | 0.00 | -0.12 | 0.12 | 0.98 | 0.01 | -0.04 | 0.06 | 0.92 |
| Maternal prenatal smoking | 0.04 | -0.02 | 0.10 | 0.40 | 0.00 | -0.02 | 0.03 | 0.92 |
| Parental education | -0.09 | -0.12 | -0.05 | 0.00 | 0.00 | -0.02 | 0.01 | 0.92 |
| Parental income | -0.01 | -0.03 | 0.01 | 0.42 | 0.00 | -0.01 | 0.01 | 0.92 |
| Maternal prenatal life events | 0.03 | 0.01 | 0.06 | 0.04 | 0.00 | 0.00 | 0.01 | 0.92 |
| Maternal adverse life events | 0.05 | 0.02 | 0.09 | 0.01 | 0.00 | -0.01 | 0.01 | 0.92 |
| Maternal postnatal depression | 0.04 | 0.01 | 0.06 | 0.02 | -0.01 | -0.02 | 0.00 | 0.86 |
| Maternal concurrent distress | 0.11 | 0.07 | 0.15 | 0.00 | 0.02 | 0.00 | 0.03 | 0.06 |
| Maternal prenatal distress | 0.01 | -0.02 | 0.05 | 0.49 | 0.00 | -0.01 | 0.01 | 0.92 |
| Maternal relationship problems | 0.07 | 0.04 | 0.10 | 0.00 | 0.00 | -0.01 | 0.00 | 0.92 |
| Paternal relationship problems | 0.02 | -0.01 | 0.04 | 0.40 | 0.00 | -0.01 | 0.01 | 0.92 |
| Paternal prenatal life events | 0.00 | -0.03 | 0.04 | 0.98 | 0.00 | -0.01 | 0.02 | 0.92 |

*Note: EST = estimate; LCI = lower 95% confidence interval; UCI = upper 95% confidence interval;
P_FDR_ = False Discovery Rate corrected p-value.*

| **Table S10: Sensitivity analysis with behavioural problems as the outcome (adjusted)** | | | | | | | | |
| --- | --- | --- | --- | --- | --- | --- | --- | --- |
|  | **Intercept** | | | | **Slope** | | | |
| **Predictor** | **EST** | **LCI** | **UCI** | **P_FDR_** | **EST** | **LCI** | **UCI** | **P_FDR_** |
| Paternal prenatal distress | 0.01 | -0.02 | 0.05 | 0.74 | 0.01 | 0.00 | 0.02 | 0.66 |
| Maternal alcohol problems | -0.01 | -0.05 | 0.03 | 0.78 | 0.00 | -0.01 | 0.01 | 0.90 |
| Maternal at-risk drinking | 0.02 | -0.03 | 0.08 | 0.73 | 0.00 | -0.02 | 0.01 | 0.90 |
| Paternal at-risk drinking | -0.03 | -0.09 | 0.03 | 0.71 | -0.01 | -0.03 | 0.01 | 0.66 |
| Paternal prenatal smoking | 0.04 | -0.01 | 0.08 | 0.44 | 0.01 | 0.00 | 0.03 | 0.49 |
| Maternal prenatal smoking | -0.01 | -0.06 | 0.04 | 0.76 | 0.00 | -0.02 | 0.02 | 0.90 |
| Parental education | 0.01 | -0.06 | 0.07 | 0.84 | 0.01 | -0.02 | 0.03 | 0.90 |
| Parental income | -0.05 | -0.09 | -0.02 | 0.10 | 0.00 | -0.02 | 0.01 | 0.90 |
| Maternal prenatal life events | -0.03 | -0.07 | 0.01 | 0.44 | -0.02 | -0.03 | 0.00 | 0.12 |
| Maternal adverse life events | 0.02 | -0.02 | 0.07 | 0.71 | 0.00 | -0.01 | 0.02 | 0.90 |
| Maternal postnatal depression | -0.04 | -0.08 | 0.00 | 0.18 | -0.02 | -0.03 | -0.01 | 0.02 |
| Maternal concurrent distress | 0.01 | -0.04 | 0.06 | 0.76 | 0.00 | -0.02 | 0.02 | 0.90 |
| Maternal prenatal distress | -0.02 | -0.06 | 0.03 | 0.75 | -0.01 | -0.02 | 0.01 | 0.66 |
| Maternal relationship problems | -0.05 | -0.10 | 0.00 | 0.18 | -0.03 | -0.05 | -0.01 | 0.00 |
| Paternal relationship problems | 0.00 | -0.04 | 0.04 | 0.90 | 0.00 | -0.01 | 0.01 | 0.90 |
| Paternal prenatal life events | 0.01 | -0.04 | 0.06 | 0.76 | 0.00 | -0.01 | 0.02 | 0.90 |

*Note: EST = estimate; LCI = lower 95% confidence interval; UCI = upper 95% confidence interval;
P_FDR_ = False Discovery Rate corrected p-value.*

| **Table S11: Sensitivity analysis with emotional problems as the outcome (unadjusted)** | | | | | | | | |
| --- | --- | --- | --- | --- | --- | --- | --- | --- |
|  | **Intercept** | | | | **Slope** | | | |
| **Predictor** | **EST** | **LCI** | **UCI** | **P_FDR_** | **EST** | **LCI** | **UCI** | **P_FDR_** |
| Paternal prenatal distress | 0.01 | -0.02 | 0.04 | 0.62 | 0.00 | -0.01 | 0.01 | 1.00 |
| Maternal alcohol problems | 0.06 | 0.00 | 0.12 | 0.12 | 0.01 | 0.00 | 0.03 | 0.23 |
| Maternal at-risk drinking | -0.02 | -0.19 | 0.16 | 0.97 | -0.01 | -0.03 | 0.00 | 0.46 |
| Paternal at-risk drinking | 0.01 | -0.59 | 0.61 | 0.99 | 0.00 | -0.06 | 0.06 | 1.00 |
| Paternal prenatal smoking | 0.00 | -0.13 | 0.13 | 0.99 | 0.00 | -0.03 | 0.03 | 1.00 |
| Maternal prenatal smoking | 0.04 | -0.03 | 0.11 | 0.47 | 0.01 | -0.01 | 0.02 | 0.74 |
| Parental education | -0.02 | -0.05 | 0.00 | 0.20 | 0.01 | 0.00 | 0.02 | 0.23 |
| Parental income | -0.02 | -0.04 | 0.00 | 0.09 | 0.00 | -0.01 | 0.01 | 1.00 |
| Maternal prenatal life events | 0.03 | 0.00 | 0.06 | 0.09 | 0.00 | -0.01 | 0.01 | 1.00 |
| Maternal adverse life events | 0.06 | 0.01 | 0.10 | 0.09 | 0.01 | 0.00 | 0.02 | 0.74 |
| Maternal postnatal depression | 0.09 | 0.06 | 0.12 | 0.00 | 0.01 | 0.00 | 0.02 | 0.23 |
| Maternal concurrent distress | 0.10 | 0.05 | 0.14 | 0.00 | 0.00 | -0.01 | 0.02 | 0.77 |
| Maternal prenatal distress | 0.07 | 0.03 | 0.11 | 0.00 | 0.00 | -0.01 | 0.01 | 1.00 |
| Maternal relationship problems | 0.03 | 0.00 | 0.06 | 0.09 | -0.01 | -0.02 | 0.00 | 0.23 |
| Paternal relationship problems | -0.01 | -0.04 | 0.01 | 0.48 | 0.00 | -0.01 | 0.00 | 0.82 |
| Paternal prenatal life events | 0.01 | -0.03 | 0.05 | 0.85 | 0.00 | -0.01 | 0.01 | 0.77 |

*Note: EST = estimate; LCI = lower 95% confidence interval; UCI = upper 95% confidence interval;
P_FDR_ = False Discovery Rate corrected p-value.*

| **Table S12: Sensitivity analysis with emotional problems as the outcome (adjusted)** | | | | | | | | |
| --- | --- | --- | --- | --- | --- | --- | --- | --- |
|  | **Intercept** | | | | **Slope** | | | |
| **Predictor** | **EST** | **LCI** | **UCI** | **P_FDR_** | **EST** | **LCI** | **UCI** | **P_FDR_** |
| Paternal prenatal distress | -0.03 | -0.06 | 0.01 | 0.33 | -0.01 | -0.02 | 0.00 | 0.56 |
| Maternal alcohol problems | 0.03 | -0.01 | 0.07 | 0.35 | 0.00 | -0.01 | 0.02 | 0.75 |
| Maternal at-risk drinking | -0.04 | -0.09 | 0.00 | 0.20 | -0.01 | -0.03 | 0.00 | 0.30 |
| Paternal at-risk drinking | -0.06 | -0.11 | -0.02 | 0.07 | -0.01 | -0.03 | 0.01 | 0.57 |
| Paternal prenatal smoking | 0.04 | -0.01 | 0.08 | 0.29 | 0.00 | -0.01 | 0.02 | 0.83 |
| Maternal prenatal smoking | 0.00 | -0.04 | 0.05 | 0.90 | 0.00 | -0.01 | 0.02 | 0.83 |
| Parental education | -0.01 | -0.07 | 0.05 | 0.87 | -0.01 | -0.04 | 0.01 | 0.64 |
| Parental income | -0.06 | -0.10 | -0.03 | 0.00 | 0.00 | -0.02 | 0.01 | 0.75 |
| Maternal prenatal life events | -0.01 | -0.04 | 0.02 | 0.74 | -0.01 | -0.02 | 0.00 | 0.30 |
| Maternal adverse life events | 0.04 | 0.01 | 0.07 | 0.10 | 0.01 | 0.00 | 0.03 | 0.30 |
| Maternal postnatal depression | 0.01 | -0.03 | 0.04 | 0.85 | 0.00 | -0.01 | 0.01 | 0.76 |
| Maternal concurrent distress | 0.00 | -0.04 | 0.04 | 0.90 | -0.01 | -0.03 | 0.00 | 0.30 |
| Maternal prenatal distress | 0.02 | -0.02 | 0.06 | 0.48 | 0.00 | -0.01 | 0.02 | 0.75 |
| Maternal relationship problems | -0.02 | -0.06 | 0.01 | 0.40 | 0.00 | -0.02 | 0.01 | 0.75 |
| Paternal relationship problems | 0.02 | -0.01 | 0.05 | 0.41 | 0.00 | -0.01 | 0.02 | 0.75 |
| Paternal prenatal life events | -0.01 | -0.06 | 0.04 | 0.88 | 0.00 | -0.02 | 0.01 | 0.83 |

*Note: EST = estimate; LCI = lower 95% confidence interval; UCI = upper 95% confidence interval;
P_FDR_ = False Discovery Rate corrected p-value.*

| **Table S13: Unweighted results from IPPW sensitivity analysis (differentiation, unadjusted)** | | | | | | | | |
| --- | --- | --- | --- | --- | --- | --- | --- | --- |
|  | **Intercept** | | | | **Slope** | | | |
| **Predictor** | **EST** | **LCI** | **UCI** | **P_FDR_** | **EST** | **LCI** | **UCI** | **P_FDR_** |
| Paternal prenatal distress | -0.01 | -0.04 | 0.01 | 0.73 | 0.00 | -0.01 | 0.01 | 0.86 |
| Maternal alcohol problems | -0.01 | -0.04 | 0.02 | 0.87 | 0.00 | -0.01 | 0.01 | 0.79 |
| Maternal at-risk drinking | 0.04 | 0.01 | 0.07 | 0.01 | 0.01 | -0.01 | 0.02 | 0.79 |
| Paternal at-risk drinking | -0.01 | -0.06 | 0.03 | 0.87 | -0.01 | -0.06 | 0.04 | 0.79 |
| Paternal prenatal smoking | 0.00 | -0.03 | 0.02 | 0.90 | 0.00 | 0.00 | 0.01 | 0.79 |
| Maternal prenatal smoking | 0.00 | -0.03 | 0.03 | 0.93 | 0.00 | -0.01 | 0.01 | 0.79 |
| Parental education | -0.06 | -0.08 | -0.03 | 0.00 | -0.01 | -0.02 | 0.00 | 0.02 |
| Parental income | 0.01 | -0.01 | 0.03 | 0.73 | 0.00 | -0.01 | 0.01 | 0.86 |
| Maternal prenatal life events | 0.00 | -0.02 | 0.03 | 0.87 | 0.00 | -0.01 | 0.01 | 0.79 |
| Maternal adverse life events | 0.00 | -0.03 | 0.02 | 0.90 | 0.00 | -0.01 | 0.01 | 0.79 |
| Maternal postnatal depression | -0.05 | -0.07 | -0.02 | 0.01 | -0.01 | -0.02 | 0.00 | 0.02 |
| Maternal concurrent distress | 0.02 | -0.02 | 0.05 | 0.73 | 0.01 | 0.00 | 0.02 | 0.34 |
| Maternal prenatal distress | -0.05 | -0.09 | -0.01 | 0.03 | 0.00 | -0.02 | 0.01 | 0.79 |
| Maternal relationship problems | 0.04 | 0.01 | 0.06 | 0.01 | 0.00 | 0.00 | 0.01 | 0.71 |
| Paternal relationship problems | 0.03 | 0.01 | 0.05 | 0.03 | 0.00 | 0.00 | 0.01 | 0.64 |
| Paternal prenatal life events | -0.01 | -0.04 | 0.02 | 0.87 | 0.00 | -0.01 | 0.01 | 0.79 |

*Note: IPPW = inverse probability of participation weighting; EST = estimate; LCI = lower 95% confid-
ence interval; UCI = upper 95% confidence interval; P_FDR_ = False Discovery Rate corrected p-value.*

| **Table S14: Weighted results from IPPW sensitivity analysis (differentiation, unadjusted)** | | | | | | | | |
| --- | --- | --- | --- | --- | --- | --- | --- | --- |
|  | **Intercept** | | | | **Slope** | | | |
| **Predictor** | **EST** | **LCI** | **UCI** | **P_FDR_** | **EST** | **LCI** | **UCI** | **P_FDR_** |
| Paternal prenatal distress | -0.02 | -0.05 | 0.01 | 0.42 | 0.00 | -0.01 | 0.01 | 0.99 |
| Maternal alcohol problems | -0.01 | -0.05 | 0.02 | 0.69 | -0.01 | -0.02 | 0.01 | 0.89 |
| Maternal at-risk drinking | 0.03 | -0.04 | 0.11 | 0.69 | 0.00 | -0.02 | 0.03 | 0.91 |
| Paternal at-risk drinking | 0.00 | -0.28 | 0.28 | 0.99 | -0.01 | -0.07 | 0.05 | 0.99 |
| Paternal prenatal smoking | -0.01 | -0.06 | 0.04 | 0.86 | 0.00 | -0.01 | 0.02 | 0.89 |
| Maternal prenatal smoking | -0.01 | -0.07 | 0.04 | 0.86 | -0.01 | -0.03 | 0.01 | 0.89 |
| Parental education | -0.06 | -0.10 | -0.02 | 0.02 | -0.02 | -0.03 | 0.00 | 0.06 |
| Parental income | 0.02 | -0.01 | 0.04 | 0.45 | 0.00 | -0.01 | 0.01 | 0.99 |
| Maternal prenatal life events | 0.00 | -0.03 | 0.03 | 0.99 | 0.00 | -0.01 | 0.02 | 0.89 |
| Maternal adverse life events | 0.00 | -0.04 | 0.04 | 0.99 | 0.00 | -0.01 | 0.01 | 0.91 |
| Maternal postnatal depression | -0.05 | -0.09 | -0.02 | 0.03 | -0.02 | -0.03 | -0.01 | 0.06 |
| Maternal concurrent distress | 0.04 | -0.01 | 0.08 | 0.33 | 0.01 | 0.00 | 0.03 | 0.15 |
| Maternal prenatal distress | -0.07 | -0.12 | -0.01 | 0.09 | 0.00 | -0.02 | 0.02 | 0.99 |
| Maternal relationship problems | 0.03 | 0.00 | 0.06 | 0.11 | 0.00 | -0.01 | 0.01 | 0.89 |
| Paternal relationship problems | 0.04 | 0.01 | 0.06 | 0.03 | 0.01 | 0.00 | 0.01 | 0.49 |
| Paternal prenatal life events | -0.01 | -0.05 | 0.03 | 0.86 | 0.00 | -0.02 | 0.01 | 0.90 |

*Note: IPPW = inverse probability of participation weighting; EST = estimate; LCI = lower 95% confid-
ence interval; UCI = upper 95% confidence interval; P_FDR_ = False Discovery Rate corrected p-value.*

| **Table S15: Unweighted results from IPPW sensitivity analysis (total, unadjusted)** | | | | | | | | |
| --- | --- | --- | --- | --- | --- | --- | --- | --- |
|  | **Intercept** | | | | **Slope** | | | |
| **Predictor** | **EST** | **LCI** | **UCI** | **P_FDR_** | **EST** | **LCI** | **UCI** | **P_FDR_** |
| Paternal prenatal distress | 0.00 | -0.02 | 0.03 | 0.97 | 0.00 | -0.01 | 0.01 | 0.99 |
| Maternal alcohol problems | 0.07 | 0.00 | 0.13 | 0.09 | 0.01 | 0.00 | 0.03 | 0.47 |
| Maternal at-risk drinking | 0.01 | -0.15 | 0.17 | 1.00 | -0.01 | -0.04 | 0.02 | 0.94 |
| Paternal at-risk drinking | 0.00 | -0.58 | 0.58 | 1.00 | -0.01 | -0.12 | 0.11 | 0.99 |
| Paternal prenatal smoking | 0.00 | -0.11 | 0.11 | 1.00 | 0.00 | -0.03 | 0.04 | 0.99 |
| Maternal prenatal smoking | 0.05 | -0.01 | 0.11 | 0.21 | 0.01 | -0.01 | 0.02 | 0.94 |
| Parental education | -0.07 | -0.10 | -0.04 | 0.00 | 0.00 | -0.01 | 0.01 | 0.94 |
| Parental income | -0.02 | -0.04 | 0.00 | 0.08 | 0.00 | -0.01 | 0.01 | 0.99 |
| Maternal prenatal life events | 0.04 | 0.01 | 0.07 | 0.02 | 0.00 | 0.00 | 0.01 | 0.94 |
| Maternal adverse life events | 0.06 | 0.03 | 0.10 | 0.00 | 0.00 | -0.01 | 0.01 | 0.94 |
| Maternal postnatal depression | 0.08 | 0.05 | 0.11 | 0.00 | 0.00 | -0.01 | 0.01 | 0.99 |
| Maternal concurrent distress | 0.13 | 0.08 | 0.17 | 0.00 | 0.01 | 0.00 | 0.02 | 0.47 |
| Maternal prenatal distress | 0.05 | 0.02 | 0.09 | 0.01 | 0.00 | -0.01 | 0.01 | 0.99 |
| Maternal relationship problems | 0.06 | 0.04 | 0.09 | 0.00 | -0.01 | -0.01 | 0.00 | 0.47 |
| Paternal relationship problems | 0.00 | -0.02 | 0.02 | 1.00 | 0.00 | -0.01 | 0.01 | 0.99 |
| Paternal prenatal life events | 0.01 | -0.03 | 0.05 | 0.97 | 0.00 | -0.01 | 0.01 | 0.94 |

*Note: IPPW = inverse probability of participation weighting; EST = estimate; LCI = lower 95% confid-
ence interval; UCI = upper 95% confidence interval; P_FDR_ = False Discovery Rate corrected p-value.*

| **Table S16: Weighted results from IPPW sensitivity analysis (total, unadjusted)** | | | | | | | | |
| --- | --- | --- | --- | --- | --- | --- | --- | --- |
|  | **Intercept** | | | | **Slope** | | | |
| **Predictor** | **EST** | **LCI** | **UCI** | **P_FDR_** | **EST** | **LCI** | **UCI** | **P_FDR_** |
| Paternal prenatal distress | 0.00 | -0.04 | 0.03 | 1.00 | 0.00 | -0.01 | 0.01 | 0.99 |
| Maternal alcohol problems | 0.09 | 0.00 | 0.17 | 0.14 | 0.02 | -0.03 | 0.08 | 0.99 |
| Maternal at-risk drinking | 0.00 | -0.20 | 0.21 | 1.00 | -0.01 | -0.04 | 0.02 | 0.99 |
| Paternal at-risk drinking | 0.00 | -0.69 | 0.69 | 1.00 | -0.01 | -0.05 | 0.04 | 0.99 |
| Paternal prenatal smoking | 0.00 | -0.10 | 0.11 | 1.00 | 0.00 | -0.01 | 0.02 | 0.99 |
| Maternal prenatal smoking | 0.05 | -0.05 | 0.15 | 0.48 | 0.01 | -0.01 | 0.02 | 0.99 |
| Parental education | -0.06 | -0.09 | -0.03 | 0.00 | 0.00 | -0.01 | 0.01 | 0.99 |
| Parental income | -0.02 | -0.06 | 0.01 | 0.24 | 0.00 | -0.01 | 0.01 | 0.99 |
| Maternal prenatal life events | 0.03 | -0.01 | 0.06 | 0.24 | 0.00 | -0.01 | 0.01 | 0.99 |
| Maternal adverse life events | 0.07 | 0.02 | 0.12 | 0.02 | 0.00 | -0.01 | 0.01 | 0.99 |
| Maternal postnatal depression | 0.07 | 0.03 | 0.12 | 0.01 | 0.00 | -0.01 | 0.01 | 0.99 |
| Maternal concurrent distress | 0.14 | 0.10 | 0.19 | 0.00 | 0.02 | 0.00 | 0.04 | 0.26 |
| Maternal prenatal distress | 0.05 | 0.01 | 0.10 | 0.07 | 0.00 | -0.02 | 0.01 | 0.99 |
| Maternal relationship problems | 0.06 | 0.02 | 0.11 | 0.03 | -0.01 | -0.02 | 0.00 | 0.78 |
| Paternal relationship problems | 0.00 | -0.02 | 0.03 | 0.95 | 0.00 | -0.01 | 0.01 | 0.99 |
| Paternal prenatal life events | 0.01 | -0.04 | 0.06 | 0.95 | 0.00 | -0.01 | 0.02 | 0.99 |

*Note: IPPW = inverse probability of participation weighting; EST = estimate; LCI = lower 95% confid-
ence interval; UCI = upper 95% confidence interval; P_FDR_ = False Discovery Rate corrected p-value.*

| **Table S17: Unweighted results from IPPW sensitivity analysis (differentiation, adjusted)** | | | | | | | | |
| --- | --- | --- | --- | --- | --- | --- | --- | --- |
|  | **Intercept** | | | | **Slope** | | | |
| **Predictor** | **EST** | **LCI** | **UCI** | **P_FDR_** | **EST** | **LCI** | **UCI** | **P_FDR_** |
| Paternal prenatal distress | 0.04 | 0.00 | 0.08 | 0.30 | 0.02 | 0.00 | 0.03 | 0.17 |
| Maternal alcohol problems | -0.03 | -0.07 | 0.01 | 0.53 | 0.00 | -0.02 | 0.01 | 0.77 |
| Maternal at-risk drinking | 0.06 | 0.01 | 0.11 | 0.30 | 0.01 | -0.01 | 0.03 | 0.59 |
| Paternal at-risk drinking | 0.02 | -0.09 | 0.13 | 0.78 | 0.00 | -0.04 | 0.04 | 0.92 |
| Paternal prenatal smoking | 0.00 | -0.06 | 0.05 | 0.90 | 0.01 | -0.01 | 0.03 | 0.59 |
| Maternal prenatal smoking | -0.01 | -0.07 | 0.04 | 0.78 | 0.00 | -0.02 | 0.01 | 0.92 |
| Parental education | 0.01 | -0.06 | 0.09 | 0.78 | 0.02 | -0.01 | 0.04 | 0.59 |
| Parental income | 0.01 | -0.03 | 0.05 | 0.78 | 0.00 | -0.01 | 0.02 | 0.92 |
| Maternal prenatal life events | -0.01 | -0.06 | 0.03 | 0.78 | 0.00 | -0.02 | 0.01 | 0.92 |
| Maternal adverse life events | -0.01 | -0.06 | 0.04 | 0.78 | -0.01 | -0.03 | 0.01 | 0.59 |
| Maternal postnatal depression | -0.04 | -0.09 | 0.00 | 0.32 | -0.01 | -0.03 | 0.00 | 0.28 |
| Maternal concurrent distress | 0.02 | -0.03 | 0.07 | 0.78 | 0.01 | 0.00 | 0.03 | 0.36 |
| Maternal prenatal distress | -0.03 | -0.08 | 0.01 | 0.49 | -0.01 | -0.03 | 0.00 | 0.36 |
| Maternal relationship problems | -0.02 | -0.06 | 0.03 | 0.78 | -0.02 | -0.04 | 0.00 | 0.17 |
| Paternal relationship problems | -0.03 | -0.06 | 0.01 | 0.53 | -0.01 | -0.02 | 0.01 | 0.59 |
| Paternal prenatal life events | 0.01 | -0.04 | 0.06 | 0.78 | 0.00 | -0.02 | 0.02 | 0.92 |

*Note: IPPW = inverse probability of participation weighting; EST = estimate; LCI = lower 95% confid-
ence interval; UCI = upper 95% confidence interval; P_FDR_ = False Discovery Rate corrected p-value.*

| **Table S18: Weighted results from IPPW sensitivity analysis (differentiation, adjusted)** | | | | | | | | |
| --- | --- | --- | --- | --- | --- | --- | --- | --- |
|  | **Intercept** | | | | **Slope** | | | |
| **Predictor** | **EST** | **LCI** | **UCI** | **P_FDR_** | **EST** | **LCI** | **UCI** | **P_FDR_** |
| Paternal prenatal distress | 0.05 | 0.00 | 0.09 | 0.57 | 0.02 | 0.00 | 0.04 | 0.14 |
| Maternal alcohol problems | -0.02 | -0.07 | 0.04 | 0.81 | 0.00 | -0.02 | 0.02 | 0.98 |
| Maternal at-risk drinking | 0.05 | -0.01 | 0.12 | 0.57 | 0.01 | -0.01 | 0.03 | 0.95 |
| Paternal at-risk drinking | 0.01 | -0.14 | 0.17 | 0.93 | 0.00 | -0.06 | 0.05 | 0.98 |
| Paternal prenatal smoking | -0.01 | -0.06 | 0.05 | 0.93 | 0.01 | -0.01 | 0.03 | 0.73 |
| Maternal prenatal smoking | 0.00 | -0.07 | 0.06 | 0.93 | 0.00 | -0.02 | 0.02 | 0.98 |
| Parental education | 0.01 | -0.08 | 0.09 | 0.93 | 0.01 | -0.02 | 0.04 | 0.73 |
| Parental income | 0.00 | -0.04 | 0.05 | 0.93 | 0.00 | -0.02 | 0.02 | 0.98 |
| Maternal prenatal life events | -0.01 | -0.06 | 0.03 | 0.81 | 0.00 | -0.02 | 0.01 | 0.98 |
| Maternal adverse life events | -0.03 | -0.08 | 0.02 | 0.61 | -0.02 | -0.04 | 0.00 | 0.25 |
| Maternal postnatal depression | -0.04 | -0.09 | 0.01 | 0.61 | -0.02 | -0.03 | 0.00 | 0.25 |
| Maternal concurrent distress | 0.04 | -0.03 | 0.12 | 0.61 | 0.03 | 0.00 | 0.06 | 0.27 |
| Maternal prenatal distress | -0.03 | -0.09 | 0.03 | 0.61 | -0.01 | -0.04 | 0.01 | 0.70 |
| Maternal relationship problems | -0.05 | -0.11 | 0.01 | 0.57 | -0.03 | -0.05 | -0.01 | 0.08 |
| Paternal relationship problems | -0.02 | -0.06 | 0.03 | 0.81 | -0.01 | -0.02 | 0.01 | 0.73 |
| Paternal prenatal life events | 0.02 | -0.04 | 0.08 | 0.81 | 0.00 | -0.02 | 0.02 | 0.98 |

*Note: IPPW = inverse probability of participation weighting; EST = estimate; LCI = lower 95% confid-
ence interval; UCI = upper 95% confidence interval; P_FDR_ = False Discovery Rate corrected p-value.*

| **Table S19: Unweighted results from IPPW sensitivity analysis (total, adjusted)** | | | | | | | | |
| --- | --- | --- | --- | --- | --- | --- | --- | --- |
|  | **Intercept** | | | | **Slope** | | | |
| **Predictor** | **EST** | **LCI** | **UCI** | **P_FDR_** | **EST** | **LCI** | **UCI** | **P_FDR_** |
| Paternal prenatal distress | -0.02 | -0.05 | 0.01 | 0.45 | 0.00 | -0.02 | 0.01 | 0.80 |
| Maternal alcohol problems | 0.00 | -0.04 | 0.04 | 1.00 | 0.00 | -0.01 | 0.01 | 0.98 |
| Maternal at-risk drinking | -0.02 | -0.07 | 0.03 | 0.55 | -0.01 | -0.03 | 0.00 | 0.34 |
| Paternal at-risk drinking | 0.06 | -0.03 | 0.14 | 0.41 | 0.01 | -0.02 | 0.04 | 0.80 |
| Paternal prenatal smoking | 0.04 | 0.00 | 0.08 | 0.20 | 0.01 | -0.01 | 0.02 | 0.59 |
| Maternal prenatal smoking | 0.00 | -0.05 | 0.04 | 1.00 | 0.00 | -0.02 | 0.02 | 0.98 |
| Parental education | 0.01 | -0.05 | 0.07 | 1.00 | 0.00 | -0.02 | 0.02 | 0.98 |
| Parental income | -0.06 | -0.10 | -0.03 | 0.02 | 0.00 | -0.02 | 0.01 | 0.83 |
| Maternal prenatal life events | -0.04 | -0.08 | 0.00 | 0.25 | -0.02 | -0.03 | 0.00 | 0.07 |
| Maternal adverse life events | 0.04 | -0.01 | 0.09 | 0.25 | 0.01 | -0.01 | 0.03 | 0.66 |
| Maternal postnatal depression | -0.02 | -0.06 | 0.01 | 0.41 | -0.02 | -0.03 | 0.00 | 0.07 |
| Maternal concurrent distress | 0.00 | -0.05 | 0.04 | 1.00 | -0.01 | -0.03 | 0.00 | 0.34 |
| Maternal prenatal distress | 0.00 | -0.04 | 0.04 | 1.00 | 0.00 | -0.02 | 0.01 | 0.83 |
| Maternal relationship problems | -0.05 | -0.09 | -0.01 | 0.16 | -0.02 | -0.04 | -0.01 | 0.05 |
| Paternal relationship problems | 0.04 | 0.00 | 0.07 | 0.17 | 0.01 | 0.00 | 0.02 | 0.34 |
| Paternal prenatal life events | 0.03 | -0.02 | 0.07 | 0.45 | 0.00 | -0.01 | 0.02 | 0.80 |

*Note: IPPW = inverse probability of participation weighting; EST = estimate; LCI = lower 95% confid-
ence interval; UCI = upper 95% confidence interval; P_FDR_ = False Discovery Rate corrected p-value.*

| **Table S20: Weighted results from IPPW sensitivity analysis (total, adjusted)** | | | | | | | | |
| --- | --- | --- | --- | --- | --- | --- | --- | --- |
|  | **Intercept** | | | | **Slope** | | | |
| **Predictor** | **EST** | **LCI** | **UCI** | **P_FDR_** | **EST** | **LCI** | **UCI** | **P_FDR_** |
| Paternal prenatal distress | -0.02 | -0.06 | 0.02 | 0.64 | 0.00 | -0.02 | 0.01 | 0.98 |
| Maternal alcohol problems | 0.00 | -0.05 | 0.05 | 0.93 | 0.00 | -0.02 | 0.02 | 0.98 |
| Maternal at-risk drinking | -0.01 | -0.07 | 0.06 | 0.83 | -0.01 | -0.03 | 0.01 | 0.90 |
| Paternal at-risk drinking | 0.05 | -0.09 | 0.18 | 0.64 | 0.01 | -0.04 | 0.06 | 0.98 |
| Paternal prenatal smoking | 0.05 | 0.00 | 0.10 | 0.21 | 0.01 | 0.00 | 0.03 | 0.70 |
| Maternal prenatal smoking | -0.03 | -0.10 | 0.05 | 0.64 | -0.01 | -0.04 | 0.02 | 0.98 |
| Parental education | 0.01 | -0.06 | 0.09 | 0.78 | -0.01 | -0.04 | 0.03 | 0.98 |
| Parental income | -0.06 | -0.10 | -0.02 | 0.06 | 0.00 | -0.02 | 0.02 | 0.98 |
| Maternal prenatal life events | -0.04 | -0.08 | 0.01 | 0.32 | -0.02 | -0.03 | 0.00 | 0.14 |
| Maternal adverse life events | 0.04 | -0.01 | 0.09 | 0.44 | 0.00 | -0.02 | 0.03 | 0.98 |
| Maternal postnatal depression | -0.03 | -0.07 | 0.02 | 0.60 | -0.02 | -0.03 | 0.00 | 0.14 |
| Maternal concurrent distress | 0.03 | -0.05 | 0.12 | 0.64 | 0.00 | -0.04 | 0.04 | 0.98 |
| Maternal prenatal distress | -0.02 | -0.08 | 0.04 | 0.64 | -0.01 | -0.03 | 0.01 | 0.91 |
| Maternal relationship problems | -0.06 | -0.12 | 0.00 | 0.21 | -0.03 | -0.05 | 0.00 | 0.14 |
| Paternal relationship problems | 0.03 | -0.01 | 0.07 | 0.32 | 0.01 | -0.01 | 0.02 | 0.80 |
| Paternal prenatal life events | 0.03 | -0.03 | 0.09 | 0.64 | 0.01 | -0.01 | 0.03 | 0.98 |

*Note: IPPW = inverse probability of participation weighting; EST = estimate; LCI = lower 95% confid-
ence interval; UCI = upper 95% confidence interval; P_FDR_ = False Discovery Rate corrected p-value.*

## Supporting Figures


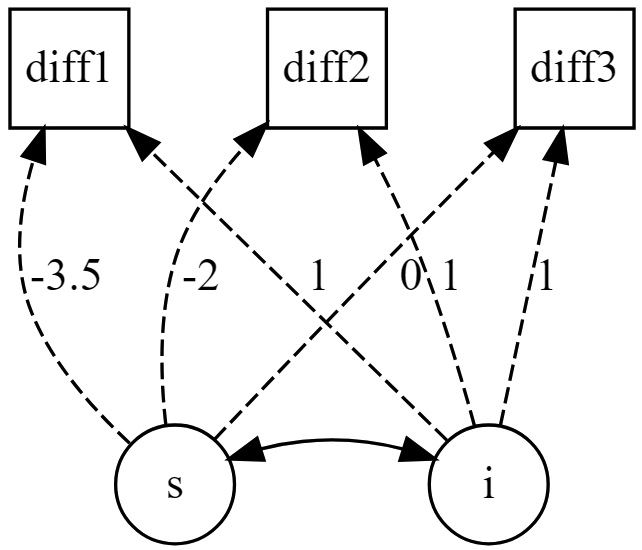


**Figure S1.** **Basic latent growth model (LGM) from age 1.5 to 5 years, with intercept at 5 years.**

*Note: boxes represent observed variables and circles represent estimated latent variables; s = slope factor with loadings -3.5, -1.5 and 0, corresponding to the temporal distance from the last wave of measurement; i = intercept factor, which loads equally on observed variables at all waves; diff1, diff2, diff3 = difference scores of behavioural and emotional problems at each wave of measurement (at 1.5 years, 3 years and 5 years of age).*

**
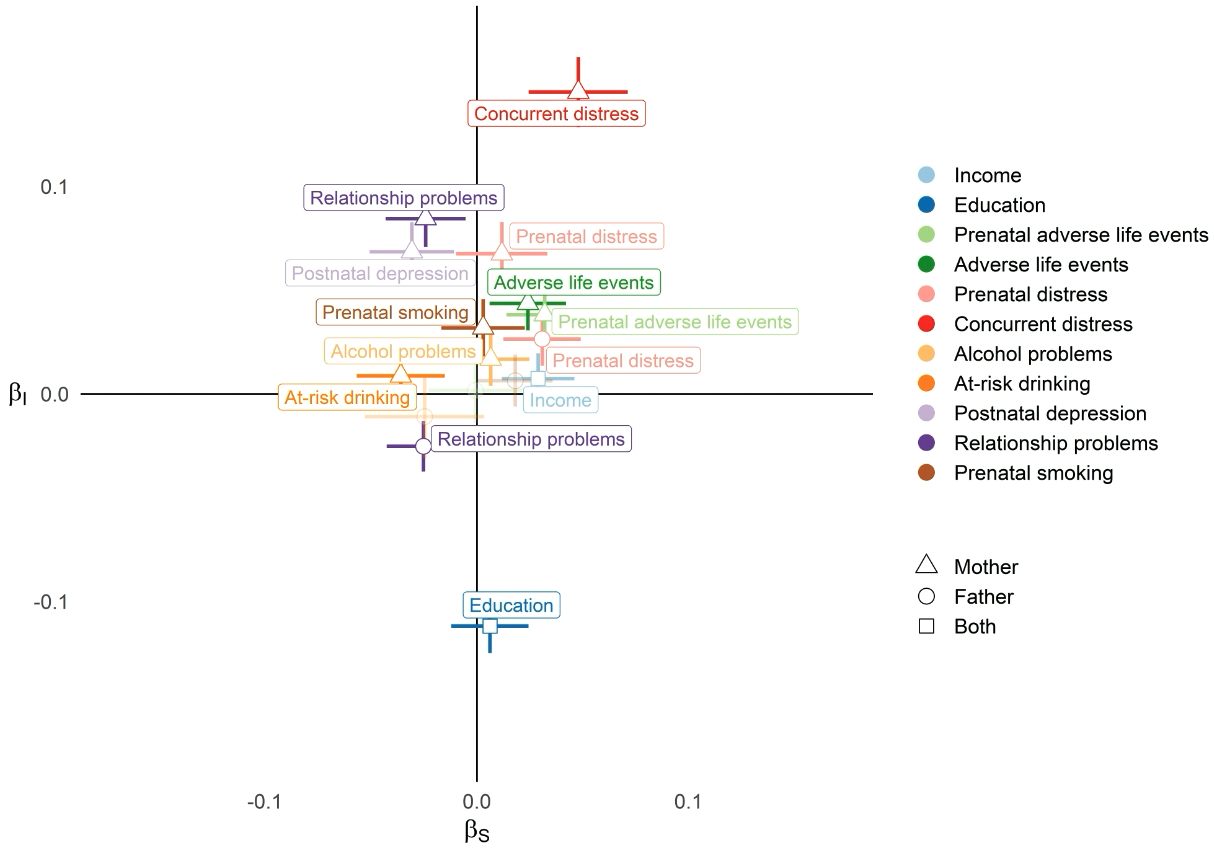
**

**Figure S2. Observational results of predictors on total behavioural and emotional problems.***Note: standardised betas for full sample observational associations between early life exposures and the intercept (β_I_) and slope (β_S_) of total behavioural and emotional problems; predictors that are labelled in the plot were significant after False Discovery Rate correction, and predictors that are transparent did not; lines denote 95% confidence intervals.*

**
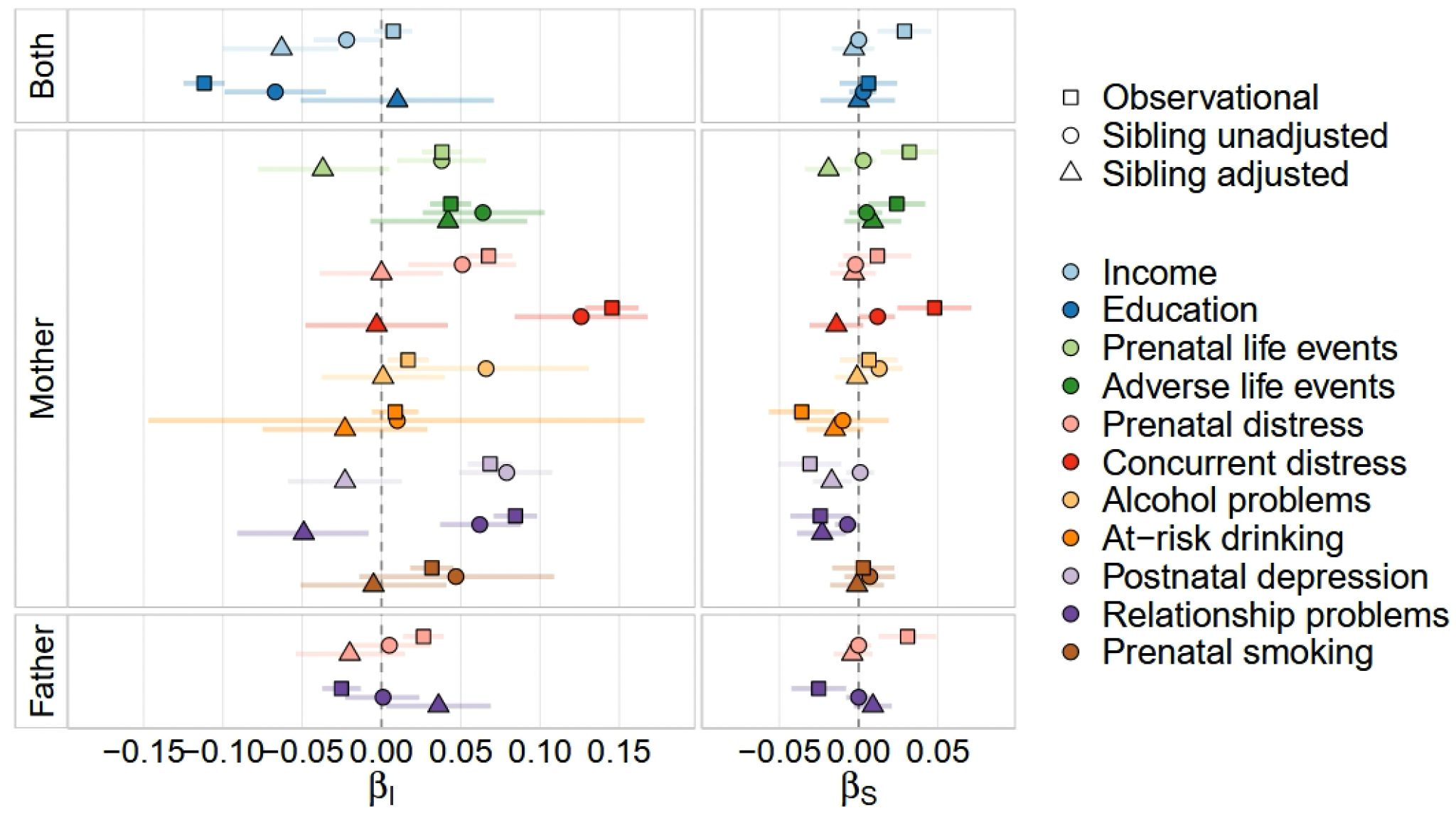
Figure S3. Full sample, sibling unadjusted and adjusted associations between exposures and total behavioural and emotional problems.***Note: standardised betas for full sample observational (N = 78,982), sibling unadjusted and adjusted (N = 23,945) associations between early life exposures and the intercept (β_I_) and slope (β_S_) of total behavioural and emotional problems during early childhood; note that the sibling adjusted results are more imprecise due to the weighting procedure used to adjust for differences between siblings and singletons, meaning that attention should mainly be paid to the attenuation (or not) of the point estimates.*

**
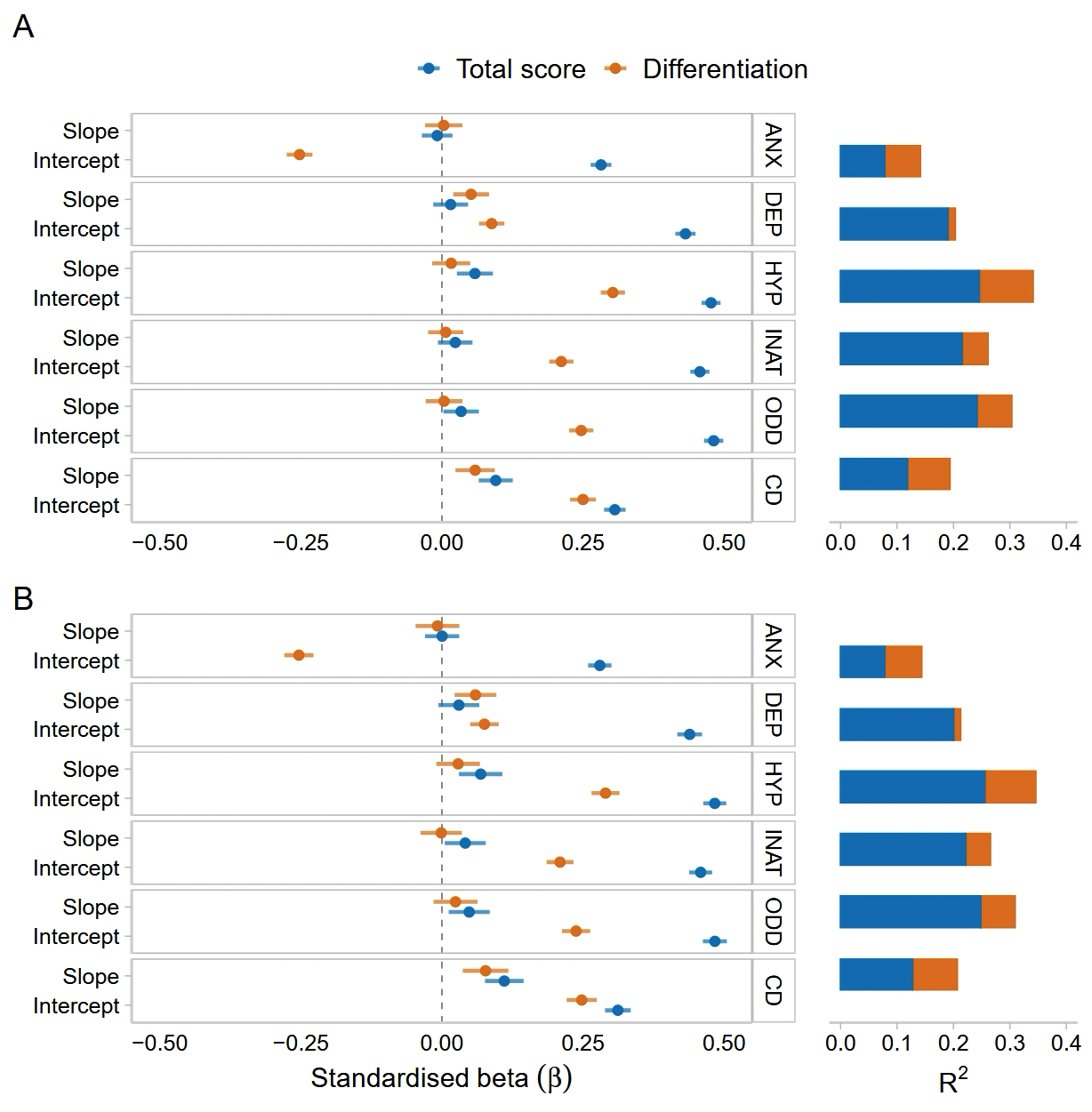
Figure S4. Results from validation analysis where differentiation and total problems in early childhood predict levels of 8-year symptoms, before and after inverse probability weighting.***Note: standardised betas* ***A****) before weighting and* ***B****) after weighting show minimal differences after accounting for potential bias from non-random non-participation; note that in this sensitivity analysis, we employed listwise deletion due to software limitations preventing the use of full information maximum likelihood when incorporating weights;* *ANX = anxiety; DEP = depression; HYP = hyperactivity; INAT = inattention; ODD = oppositional defiant disorder; CD = conduct disorder; R^2^ = R squared.*

**
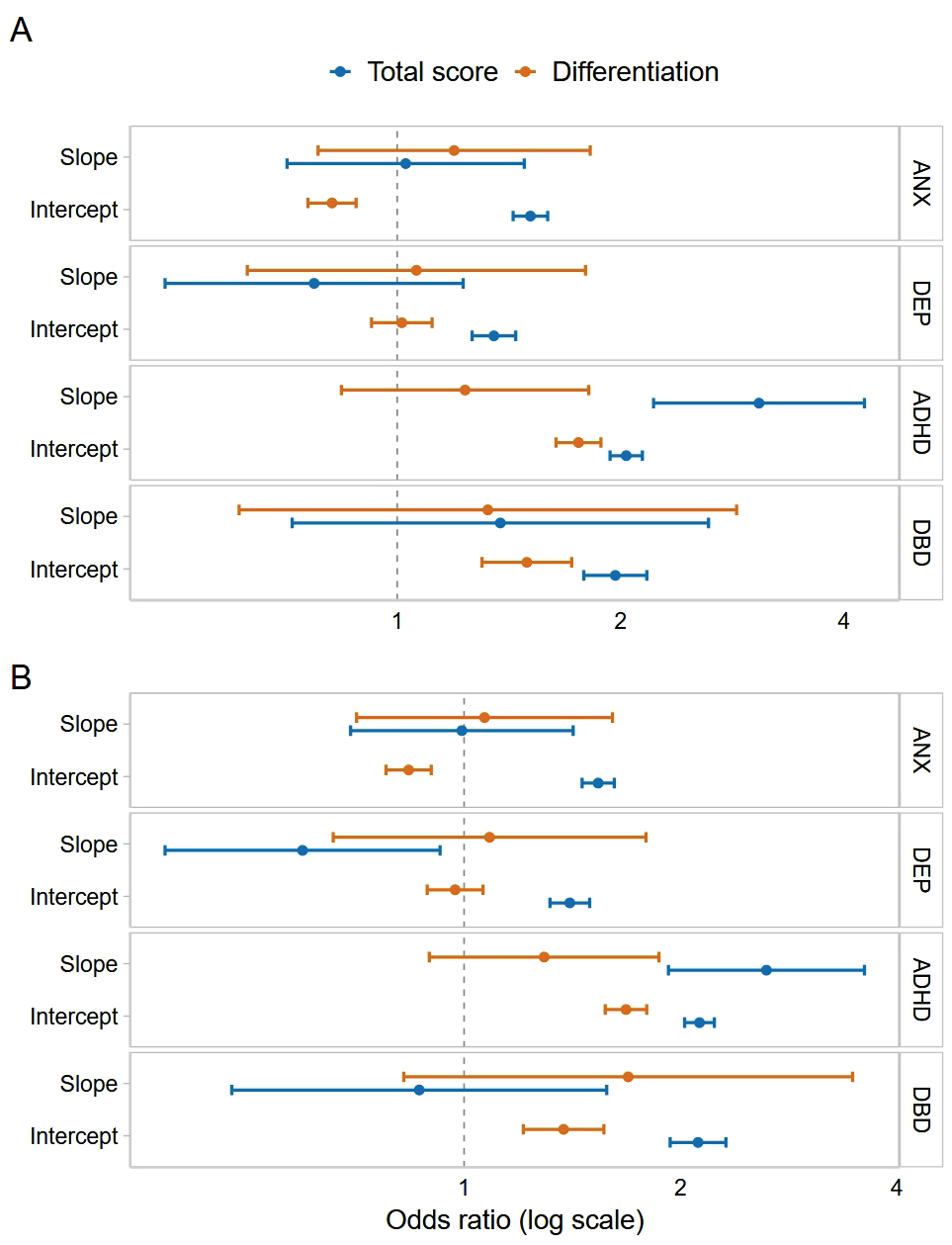
Figure S5. Validation analysis where differentiation and total problems in early childhood predict the odds of receiving diagnoses later, before and after inverse probability weighting.***Note: odds ratios* ***A****) before weighting and* ***B****) after weighting show some but overall minimal differences after accounting for bias from non-random non-participation; estimates were derived based on extracted factor scores, predicting diagnoses of mental health conditions (after age 8) in logistic regressions; ANX = anxiety; DEP = depression; ADHD = attention-deficit hyperactivity disorder; DBD = disruptive behaviour disorders.*

*
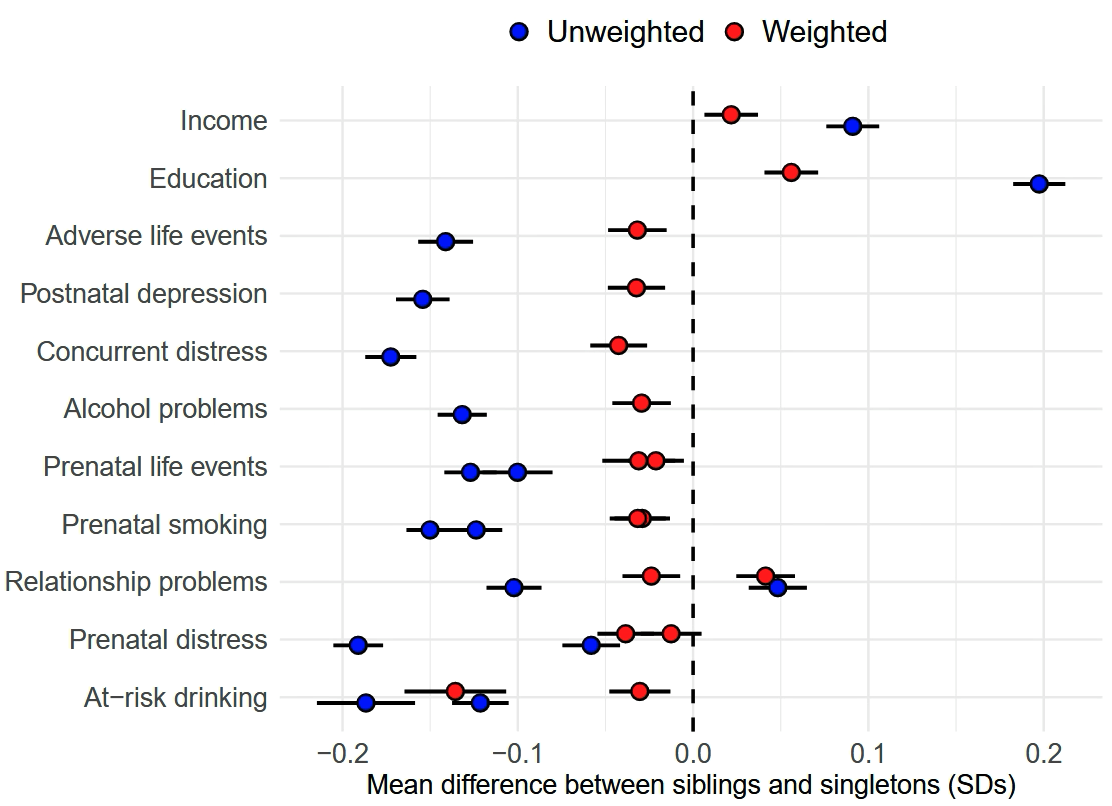
***Figure S6. Differences in predictors between siblings and singletons before/after weighting.**  *Note: mean differences in the level of each predictor before and after inverse probability of sibling weighting; most differences are notably attenuated, except for paternal at-risk drinking and relationship problems.*
